# Engineering peroxygenases for pharmaceutical applications: recent advances and future perspectives

**DOI:** 10.1039/d6sc04536g

**Published:** 2026-10-13

**Authors:** Lilin Feng, Atul Kumar, Ulrich Schwaneberg, Marisa Sárria Pereira de Passos

**Affiliations:** a Lehrstuhl für Biotechnologie, RWTH Aachen University Worringerweg 3 52074 Aachen Germany l.feng@biotec.rwth-aachen.de m.sarria@biotec.rwth-aachen.de

## Abstract

Selective oxidation is a cornerstone process in pharmaceutical biosynthesis, as it enables the conversion of inert C–H bonds into valuable functional groups such as alcohols, ketones, epoxides, and carboxylic acids, which serve as important intermediates in drug synthesis. Peroxygenases, including unspecific peroxygenases and P450 peroxygenases, have emerged as powerful biocatalysts for enzymatic oxidation, with growing applications in metabolite synthesis, late-stage C–H functionalization, and the preparation of enantioenriched intermediates for pharmaceutical development. In this review, we present the latest advances in peroxygenases engineering for pharmaceutical applications. We summarize pharmaceutical drug candidates that can serve as substrates for peroxygenases, discuss the enzymatic oxidation pathways mediated by peroxygenases for these substrates, and outline protein engineering strategies employed to improve the catalytic performance and properties of peroxygenases in pharmaceutical biosynthesis. We also discuss the potential of emerging artificial intelligence (AI) strategies for multi-dimensional peroxygenase engineering including catalytic activity, thermostability, hydrogen peroxide tolerance, heterologous expression, substrate scope, regioselectivity, and enantioselectivity. Overall, continued progress in peroxygenase engineering will drive the transformation of these biocatalysts for versatile industrial applications, thereby enabling sustainable drug synthesis, efficient metabolite production, and innovative therapeutic strategies.

## Abstract 2

Engineering peroxygenases for sustainable pharmaceutical oxyfunctionalization.

## Graphical abstract

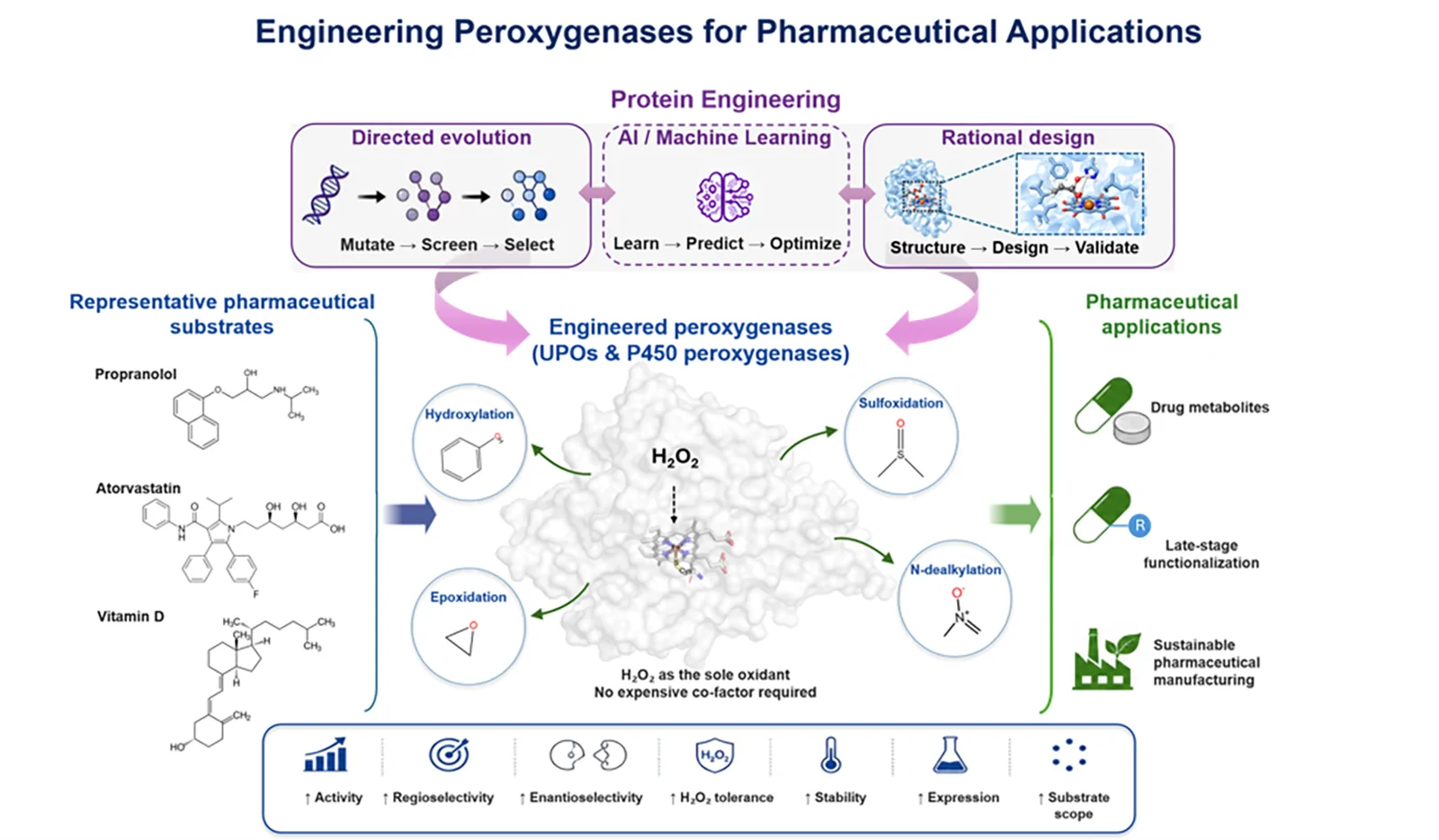

## 1. Introduction

The pharmaceutical industry increasingly relies on biocatalysis to meet the growing demand for sustainable, efficient, and selective synthetic processes. Among the various biocatalysts, heme-thiolate oxygenases have emerged as a highly promising and mechanistically diverse class of enzymes for oxyfunctionalization reactions, encompassing O_2_/NAD(P)H-dependent systems such as cytochrome P450 monooxygenases and H_2_O_2_-dependent enzymes such as unspecific peroxygenases (UPOs).^1^ In recent years, the application of peroxygenases has expanded from fundamental biochemical studies to practical roles in drug metabolism and the synthesis of pharmaceutically relevant compounds.^2^ In order to tackle their potential challenges related to enzyme expression, substrate specificity, and catalytic efficiency, protein engineering has become an important tool. Overcoming these limitations, it enables the tailoring of peroxygenases' properties to meet specific pharmaceutical requirements.

### 1.1 Biosynthesis of pharmaceuticals by (selective) oxidation

Selective oxidation is central to pharmaceutical synthesis, as it enables the conversion of inert C–H bonds into functional groups such as alcohols, ketones, epoxides, and carboxylic acids, which serve as key intermediates in active pharmaceutical ingredient (API) synthesis.^3–5^ These functionalities also provide strategic entry points for downstream derivatization and late-stage functionalization, both of which are critical for modulating potency, selectivity, and pharmacokinetic properties.^3,6^ Because biological activity is often highly sensitive to oxidation state and substitution pattern, selective oxidation frequently dictates the overall efficiency and sustainability of pharmaceutical manufacturing routes. Although conventional chemical oxidation remains widely used, its inherent limitations, including harsh reaction conditions, poor regio-/stereoselectivity, and significant environmental impact, have driven the exploration of biocatalytic alternatives.

Biocatalytic oxidation provides an attractive complementary approach to conventional chemical methods. Enzymes, particularly heme-thiolate enzymes capable of catalyzing selective oxidative transformations, offer significant advantages under environmentally compatible and energy-efficient reaction conditions. Representative examples of this enzyme class include cytochrome P450 monooxygenases (CYPs), chloroperoxidases (CPOs), and peroxygenases, all of which catalyze highly selective oxyfunctionalization reactions under mild, ambient conditions.^7–9^ Their intrinsic selectivity arises from precise substrate positioning within defined active sites and controlled oxygen activation pathways. As a result, enzymatic oxidation has become increasingly valuable for metabolite synthesis, late-stage C–H functionalization, and the preparation of enantioenriched intermediates in pharmaceutical development.^6,8^

Despite these advantages, many heme-thiolate enzymes often exhibit limited stability, cofactor dependence, and restricted substrate scope.^7,10^ In addition, their catalytic cycles typically require complex electron-transfer chains and cofactor regeneration strategies, adding complexity to process design. These constraints have stimulated interest in oxidative biocatalysts that combine high selectivity with operational simplicity. Peroxygenases represent a distinctive advancement in this context, which are capable of directly using hydrogen peroxide as the single oxidant, thereby eliminating the need for auxiliary redox partners or costly cofactors.^11–13^ Peroxygenases can catalyze a wide range of oxyfunctionalization reactions (*e.g.*, hydroxylation, epoxidation, and sulfoxidation) with high regio- and stereoselectivity, making them promising catalysts for pharmaceutical biosynthesis. By merging catalytic versatility with simplified reaction requirements, peroxygenases bridge characteristics of classical monooxygenases and peroxidases, offering streamlined oxidation platforms with significant industrial potential.

Looking forward, the integration of selective oxidation with protein engineering and synthetic biology will further expand the applicability of oxidative enzymes in drug synthesis. Protein engineering strategies including traditional approaches such as rational design and directed evolution, knowledge-driven frameworks such as KnowVolution,^14^ and data-driven methods including machine learning and generative AI can unlock new transformations and improve enzymes’ robustness under process conditions.^10,13^ Within this evolving framework, peroxygenases stand out as versatile platforms for enabling greener and more efficient oxidative routes in pharmaceutical manufacturing.

Despite these attractive catalytic properties, successful industrial implementation requires evaluation beyond catalytic activity and selectivity alone. Parameters such as enzyme production level, catalyst lifetime, space–time yield (STY), volumetric productivity, and overall process economics ultimately determine whether laboratory-scale biocatalysts can be translated into commercially viable manufacturing processes. Throughout this review, we therefore highlight not only advances in enzyme engineering but also the remaining challenges associated with industrial implementation.

### 1.2 Overview of pharmaceutical drug candidates as substrates of peroxygenases

The substrate scope of peroxygenases has undergone a remarkable expansion over the past decade, establishing these heme-thiolate enzymes as highly versatile oxidative biocatalysts for pharmaceutical drug candidates. Compared with conventional chemical oxidation methods, these enzymatic transformations exhibit markedly improved regioselectivity, demonstrating the potential of peroxygenases for the efficient synthesis of drug metabolites and pharmaceutical drug candidates. A comprehensive overview of pharmaceutical substrates, the peroxygenases employed, the biocatalyst sources, the corresponding enzymatic reactions, the resulting oxidative products, and the therapeutic relevance is summarized in Table 1.

**Table 1:** A summary of pharmaceutical substrates, the peroxygenases employed, their biocatalyst sources, enzymatic reactions, oxidative products, and therapeutic relevance

Pharmaceutical drug	Peroxygenases	Biocatalyst source	Enzymatic reaction	Oxidative product	Therapeutic class	Clinical use	References
Propranolol	UPO (engineered)	Fungal UPO variants	Selective 5′-hydroxylation	5′-Hydroxypropranolol	β-Adrenergic blocker	Hypertension, arrhythmias	15 and 16
Diclofenac	UPO	Fungal UPOs	Aromatic hydroxylation, oxidative dealkylation	4′-Hydroxydiclofenac, 5-hydroxydiclofenac	NSAID	Pain, inflammation	17
Naproxen	UPO	Fungal UPOs	Regioselective aromatic hydroxylation	6-OH-naproxen	NSAID	Pain, inflammation	16 and 18
Ibuprofen	UPO	Fungal UPOs	Aromatic hydroxylation	Hydroxy ibuprofen	NSAID	Pain, inflammation	18
Acetanilide derivatives	UPO	Fungal UPOs	Oxidative dealkylation	Hydroxylated/dealkylated metabolites	NSAID	Analgesic, antipyretic	18
Dextromethorphan	Evolved UPO variants	Fungal UPO variants	*O*- and *N*-Demethylation	*O*- and *N*-Demethylated metabolites	CNS drug	Antitussive	16 and 24
Tolbutamide	UPO	Fungal UPOs	Hydroxylation at metabolically relevant positions	4-Hydroxytolbutamide	CNS drug/antidiabetic	Type 2 diabetes	16 and 18
Sildenafil	UPO	Fungal UPOs	Selective oxidation within heterocyclic framework	Oxidized sildenafil derivatives	CNS drug/vasodilator	Erectile dysfunction, pulmonary hypertension	18
Oseltamivir	UPO	Fungal UPOs	Regioselective functionalization	Hydroxylated/oxidized oseltamivir	Antiviral	Influenza treatment	18
Atorvastatin	Engineered CYP102A1 variants	Bacterial CYP102A1	Hydroxylation	Hydroxyatorvastatin derivatives	Lipid-regulating/cardiovascular	Hypercholesterolemia	20
Testosterone	Fungal UPOs	Fungal UPOs	4,5β-Epoxidation (major); 16α-hydroxylation (minor)	Testosterone 4,5β-epoxide (major); 16α-hydroxytestosterone (minor)	Steroidal hormone	Hormone replacement therapy	21 and 25
Calciferols (vitamin D analogues)	Fungal UPOs	Fungal UPOs	Regio- and stereoselective oxidation	Hydroxylated vitamin D metabolites	Steroidal/terpenoid drugs	Bone health, calcium regulation	22
SAR548304	*Marasmius rotula* UPO	Fungal UPO	Selective *N*-dealkylation	Mono- and di-desmethyl metabolites	Investigational drug	Late-stage drug candidate	23

β-Adrenergic blockers represent one of the earliest and most extensively studied classes of substrates in peroxygenase biocatalysis. Among these, propranolol is commonly used as a model compound, for which engineered unspecific peroxygenases (UPOs) catalyze highly selective 5′-hydroxylation to form the human-relevant metabolite 5′-hydroxypropranolol.^15,16^ Non-steroidal anti-inflammatory drugs (NSAIDs), including diclofenac, naproxen, ibuprofen, and acetanilide derivatives, are also efficiently transformed by peroxygenases. In these UPO-mediated reactions, the enzymes catalyze regioselective aromatic hydroxylation and oxidative dealkylation, closely mimicking cytochrome P450-mediated metabolism while operating without NADPH-dependent electron-transfer systems. A representative example is the biocatalytic conversion of diclofenac into its major human metabolites, demonstrating the potential of UPOs for applications in toxicological and pharmacokinetic studies.^17,18^

The substrate scope of peroxygenases has further expanded to the central nervous system (CNS) therapeutics through protein engineering. For instance, evolved UPO variants have been applied to the biotransformation of dextromethorphan, enabling the preparative-scale formation of its *O*- and *N*-demethylated metabolites.^16^ Similarly, the sulfonylurea drug tolbutamide undergoes regioselective hydroxylation at metabolically relevant positions, while sildenafil can be selectively oxidized within its heterocyclic core. The successful peroxygenase-mediated oxidation of the antiviral prodrug oseltamivir demonstrates that complex, multifunctional pharmaceutical scaffolds are compatible with enzymatic oxidative modification.^16,18,19^

In addition, lipid-regulating and cardiovascular drugs have attracted interest as substrates for peroxygenase catalysis. Engineered CYP102A1-based peroxygenase variants catalyze the hydroxylation of atorvastatin and related statins, generating oxidized metabolites that are challenging to obtain with high selectivity through conventional chemical methods.^20^ Peroxygenases are also capable of transforming steroidal and terpenoid-derived compounds; for example, fungal UPOs catalyze site-selective hydroxylation of testosterone and related steroid frameworks, while oxidation of calciferols (vitamin D analogues) provides access to clinically relevant metabolites with high regio- and stereoselectivity.^21,22^ Furthermore, the potential of peroxygenases for late-stage functionalization is demonstrated by the selective *N*-dealkylation of the drug candidate SAR548304 catalyzed by *Marasmius rotula* UPO, producing mono- and di-desmethyl derivatives in high yields and often outperforming conventional chemical approaches.^23^

Overall, the range of pharmaceutical substrates processed by peroxygenases includes β-blockers, NSAIDs, antivirals, CNS drugs, statins, steroid hormones, and emerging therapeutic candidates. This broad substrate diversity reflects the intrinsic plasticity of the enzyme active site together with the controlled radical chemistry mediated by the heme-thiolate center. When combined with advanced protein engineering strategies, such as active-site remodeling, improved peroxide tolerance, and enhanced catalytic turnover, peroxygenases are increasingly emerging as powerful platforms for selective late-stage functionalization, metabolite synthesis, and the oxidative diversification of complex drug molecules.

## 2. Current enzymatic oxidation pathways

### 2.1 P450 peroxygenases

Cytochrome P450s (CYPs) are attractive enzyme candidates for the biosynthesis of pharmaceuticals, as these are involved in numerous biosynthetic pathways that produce natural and bioactive compounds across a wide range of organisms.^26^ Moreover, P450s represent one of the largest and most diverse enzyme superfamilies, with more than 300 000 annotated gene entries reported to date.^27^ In recent years, the continuous discovery of novel P450 enzymes, reactions, and metabolites has further highlighted their broad applicability in pharmaceutical synthesis.

Canonical P450s usually employ molecular oxygen as the oxidant and two electrons derived from nicotinamide cofactors (NAD(P)H) to catalyze the substrate monooxygenation.^28^ These enzymes catalyze a wide variety of oxidative transformations, including hydroxylation, epoxidation, dealkylation, and sulfoxidation.^29^

Unlike common P450 enzymes, certain cytochrome P450s, such as the CYP152 family, can catalyze the H_2_O_2_-dependent hydroxylation of pharmaceuticals *via* the ‘peroxide shunt’ pathway.^30–32^ This alternative pathway does not require NAD(P)H, molecular oxygen, and redox partner proteins for the generation of compound I intermediate. Cytochrome P450s capable of efficient peroxide-shunt catalysis are therefore classified as P450 peroxygenases, overcoming key limitations associated with classical P450 systems, including dependence on costly cofactors and complex redox partner systems to catalyze corresponding reactions.^33^ Although both enzyme classes share similar oxidation reactions, they differ fundamentally in their catalytic mechanisms, cofactor requirements, and practical applicability. In particular, canonical P450 monooxygenases possess a deeply buried heme center accessed through hydrophobic substrate channels and rely on dedicated reductase partners for electron transfer, features that contribute to limited coupling efficiency and the formation of reactive oxygen species (ROS) under certain conditions.^33^ The reaction mechanisms of cytochrome P450 are illustrated in Fig. 1.

**Fig. 1:**
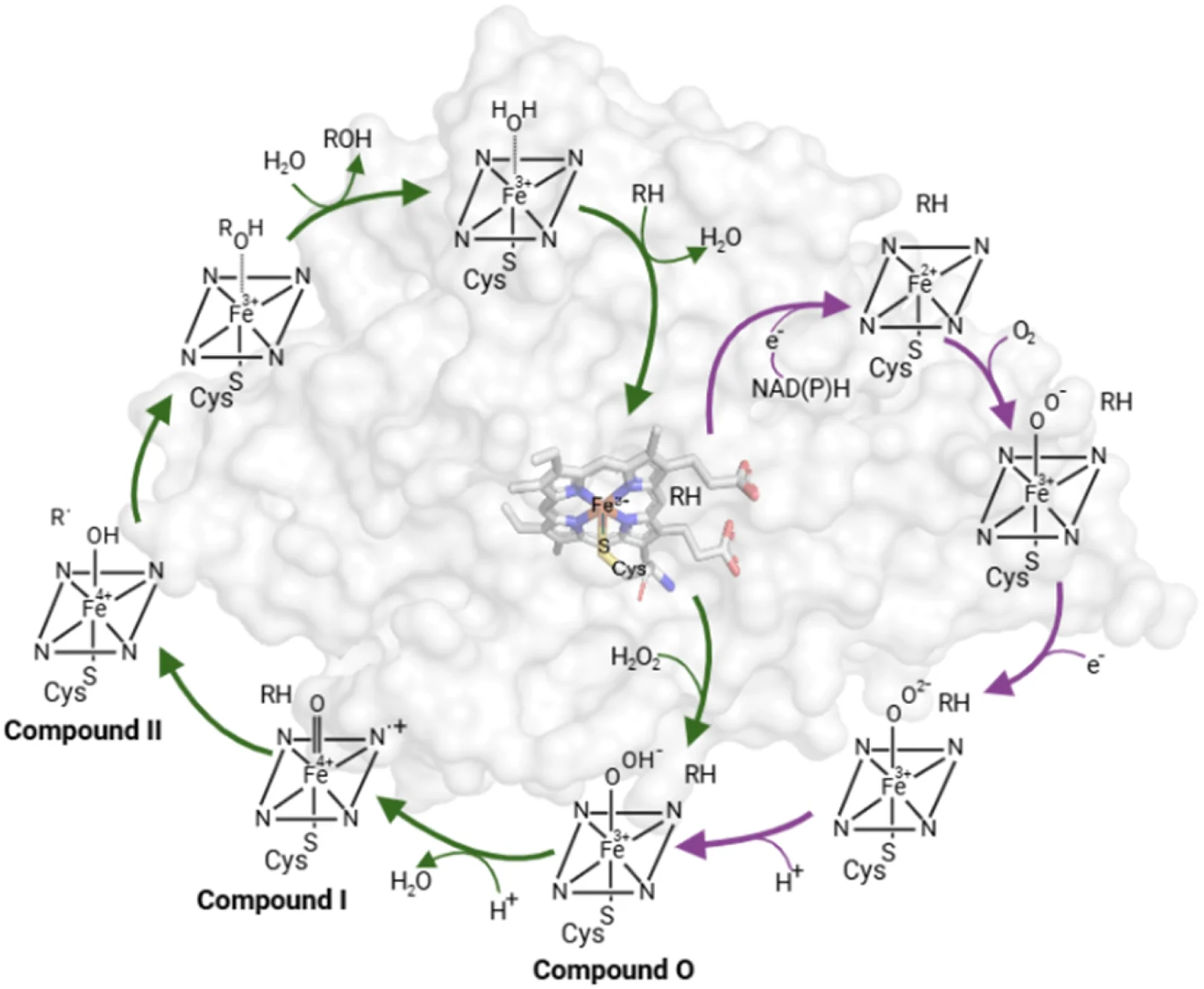
Comparison of the catalytic cycles of canonical cytochrome P450 monooxygenases and peroxygenases. Both enzyme classes converge on the reactive compound I intermediate to catalyze substrate oxygenation. The canonical P450 cycle requires O_2_ activation through sequential NAD(P)H-dependent reduction steps mediated by dedicated reductase proteins (purple route), whereas peroxygenases access compound I directly *via* heterolytic cleavage of H_2_O_2_ through the peroxide shunt (green route), bypassing the need for cofactors, electron transfer partners, and molecular oxygen. Created with https://BioRender.com.

Several naturally occurring P450 peroxygenases have been identified within the CYP152 family. Examples include CYP152A1 from *Bacillus subtilis* IFO14144 (P450_BSβ_),^30^ CYP152B1 from *Sphingomonas paucimobilis* (P450_SPα_),^34^ CYP152K6 from *Bacillus methanolicus* MGA3,^35^ CYP152L1 from *Jeotgalicoccus* sp. 8456 (OleT_JE_),^36^ and CYP152N1 from *Exiguobacterium* sp. AT1b (P450_Exα_),^37^ CYP152A2 from *Clostridium acetobutylicum* (P450_CLA_),^38^ and related homologues that catalyze H_2_O_2_-dependent oxidation of fatty acids, mainly *via* α- and β-hydroxylation, with certain members performing oxidative decarboxylation. Besides naturally occurring P450 peroxygenases, P450 monooxygenases have been converted into peroxygenases through protein engineering (discussed in Section 3.2.1).

### 2.2 Unspecific peroxygenases

Unspecific peroxygenases (UPOs, EC 1.11.2.1) are heme-thiolate enzymes that perform selective oxyfunctionalization reactions independently of cofactors, sharing a similar peroxide-driven catalytic mechanism with P450 peroxygenases. A comparison of P450 peroxygenases and UPOs is presented in Table 2. The first peroxygenase, discovered in 2004 in the basidiomycete fungus *Agrocybe aegerita* (*Aae*UPO), has since become the most extensively studied enzyme in this class, driving advances in the field due to its unique catalytic properties and remarkable substrate versatility.^39^ UPOs catalyze diverse selective oxyfunctionalization reactions, including inserting oxygen into the inert C–H and C=C bonds, as well as C–C bond cleavage, along with higher enantioselectivity and regioselectivity. Unlike cytochrome P450 monooxygenases, which depend on flavin- or iron–sulfur redox partners, UPOs require only hydrogen peroxide (H_2_O_2_) as both an electron acceptor and oxygen source. This self-sufficiency enables UPOs to mediate both one-electron oxidations (as typical peroxidases) and two-electron transfer reactions (a peroxygenase property). Catalytically, UPOs bridge the functional gap between classical peroxidases and P450s, earning their recognition as the evolutionary and mechanistic “missing link” in oxidative enzymology. Additionally, UPOs are extracellular secreted enzymes that exhibit tolerance to a wide range of pH and organic solvents, providing operational adaptability and simplicity.^40^

**Table 2:** A comparison of P450 peroxygenases and UPOs

Feature	P450 peroxygenases	Unspecific peroxygenases (UPOs)
Evolutionary origin	Derived from cytochrome P450 monooxygenases (mainly CYP152 family or engineered P450s)	Fungal heme-thiolate enzyme family
Source organism	Mainly bacteria	Predominantly fungi
Heterologous expression	Efficiently expressed in *E. coli* and yeast	Mainly secreted from fungi; heterologous expression mostly in yeast, with limited success in *E. coli*
Representative enzymes	P450_SPα_, P450_BSβ_, OleT_JE_	*Aae*UPO, *Mro*UPO, *Cgl*UPO, *Pab*UPO
Catalytic principle	Peroxide shunt pathway to generate compound I, bypassing the need for cofactors, electron transfer partners, and molecular oxygen	Dual catalytic cycle combines the “peroxide shunt” pathway of P450s and the classic peroxidase cycle
Electron donor	H_2_O_2_ (canonical P450s require NAD(P)H/O_2_)	H_2_O_2_
Reaction type	Hydroxylation, epoxidation, dealkylation, sulfoxidation, and oxidative decarboxylation (OleT)	Hydroxylation, epoxidation, *N*-oxidation, sulfoxidation, bromination, one-electron oxidation
Catalytic efficiency	Typically achieves TTNs of 10^3^–10^4^	Can achieve TTNs of >10^5^ under optimized conditions
Protein engineering focus	Improve peroxide utilization, catalytic activity, selectivity, and H_2_O_2_ tolerance	Improve heterologous expression, H_2_O_2_ tolerance, stability, and regio-/enantioselectivity
Typical pharmaceutical applications	Drug metabolite synthesis, fatty acid hydroxylation, steroid oxidation, late-stage functionalization	Drug metabolite synthesis, aromatic hydroxylation, steroid oxidation, vitamin hydroxylation, late-stage C–H functionalization

To date, over 4000 putative UPO sequences from different fungi have been deposited in genomic databases, demonstrating the natural wild diversity of UPOs.^41^ According to their structural and functional features, UPOs are phylogenetically divided into two families: short UPOs and long UPOs (Table 3). Short UPOs (such as the UPO from *Marasmius rotula* (*Mro*UPO) or the UPO from *Chaetomium globosum* (*Cgl*UPO)) are widely distributed across the fungal kingdom and are typically homodimeric proteins with molecular weights of ∼26 kDa per monomer. Their active sites feature a histidine residue as a charge stabilizer, and the two monomers are connected *via* an intermolecular disulfide bridge. In contrast, long UPOs (such as *Aae*UPO or the UPO from *Psathyrella aberdarensis* (*Pab*UPO)), found in basidiomycetes and ascomycetes, are monomeric enzymes with molecular weights of ∼44 kDa. These possess an internal disulfide bridge and use an arginine residue as the charge stabilizer at their active site.^42^ Both UPO families exhibit highly conserved active site motifs: –EHD–S–E– in short UPOs and –EGD–S–R–E– in long UPOs, as revealed by high-resolution crystal structures of *Aae*UPO and *Mro*UPO. Variations in the composition and architecture of the heme access channels between the two families determine their different substrate preferences. For example, the heme channel of short UPOs is composed of flexible aliphatic amino acids, which is shorter in length but wider, giving short UPOs a stronger affinity for larger substrates such as steroids. In contrast, the heme channel of long UPOs is composed of rigid aromatic amino acids, which determine its affinity for smaller aromatic substrates.^40,43,44^

**Table 3:** General comparison between short and long UPOs

Property	Short UPOs	Long UPOs
Molecular weight	∼29 kDa	∼44 kDa
Isoelectric point (pI)	∼6.4	∼5.8
Conformation	Dimeric	Monomeric
Disulfide bridges	Intermolecular	C-terminal
Conserved active site	–EDH–S–E–	–EGD–S–R–E–
Key stabilizing residue	His86	Arg189
Substrate scope	Bulky substrates	Small aromatic substrates
Representative examples	*Mro*UPO or *Cgl*UPO	*Aae*UPO or *Pab*UPO
Structure example	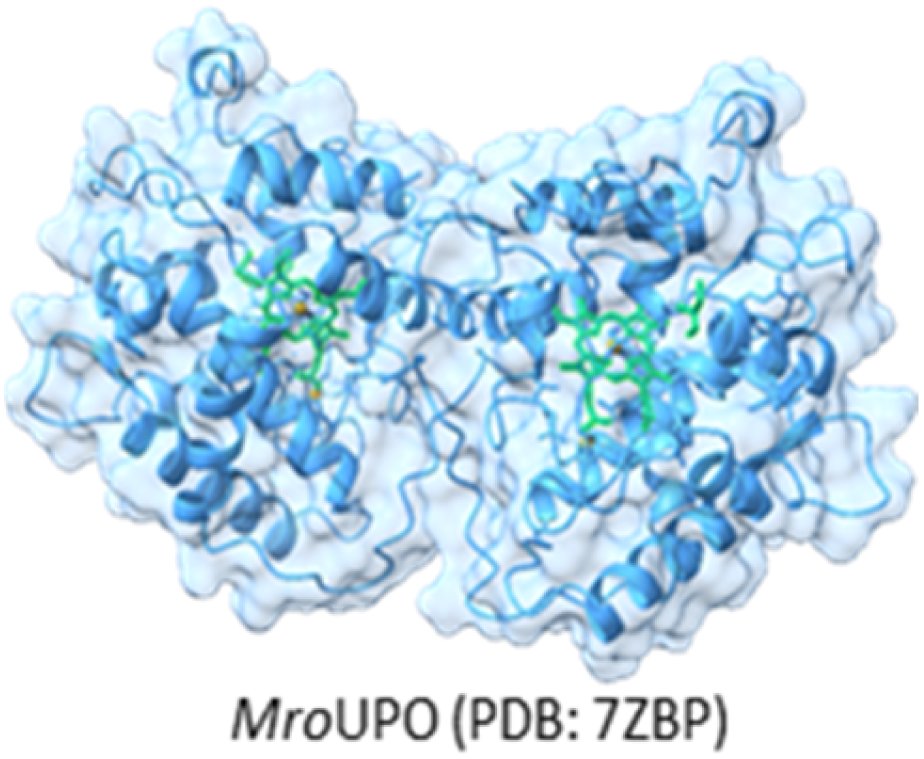	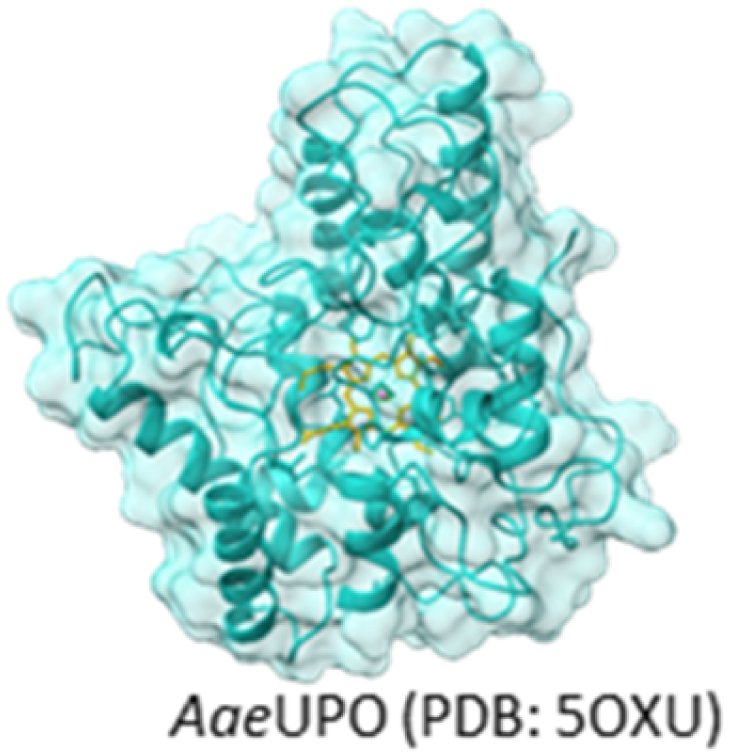

The dual catalytic cycle of UPOs combines the “peroxide shunt” pathway of P450s and the classic peroxidase cycle.^42^ The cycle begins with the UPO in its resting heme ferric state (heme-Fe^3+^ ← H_2_O). With the entry of H_2_O_2_, the H_2_O molecule is displaced, resulting in the formation of an intermediate state (heme-Fe^3+^ ← H_2_O_2_). This intermediate is deprotonated by a conserved glutamic acid (Glu196 in *Aae*UPO) to form compound 0, which decays under electron rearrangement to compound I, an active oxidized cation radical complex (^+^˙heme-Fe^4+^O=). Compound I can then follow two different routes: peroxygenase (mono(per)oxygenase) or peroxidase (route not illustrated in this review). Along the peroxygenase route, a hydrogen (H^+^ and e^−^) is abstracted from the substrate (R–H), resulting in the protonated ferryl hydroxide complex (compound II, heme-Fe^4+^–OH) and the substrate radical (R˙). Substrate radical and compound II react immediately with each other to form the hydroxylated product, while H_2_O binds again as a distal heme ligand, so the catalytic cycle starts again. The reaction mechanisms of peroxygenases are illustrated in Fig. 1 (route green).

UPOs perform several different reactions, such as hydroxylation of inactivated C–H bonds (alkanes/aromatics/benzylics), epoxidation of alkenes/aromatics, *N*-oxidation, bromination, sulfoxidation, and one-electron oxidation of phenolic compounds.^45,46^ Based on their wide range of catalytic functions, the application of UPOs for the aromatic hydroxylation of drugs to form the corresponding drug metabolites was extensively studied with long UPOs (*Aae*UPO and *Cra*UPO) since 2011. Their ability to introduce oxygen functionalities directly into complex pharmaceutical molecules in a regio- and stereoselective manner has since become a valuable approach for addressing challenges in organic synthesis. Reactions catalyzed by UPOs are summarized in Fig. 2.

**Fig. 2:**
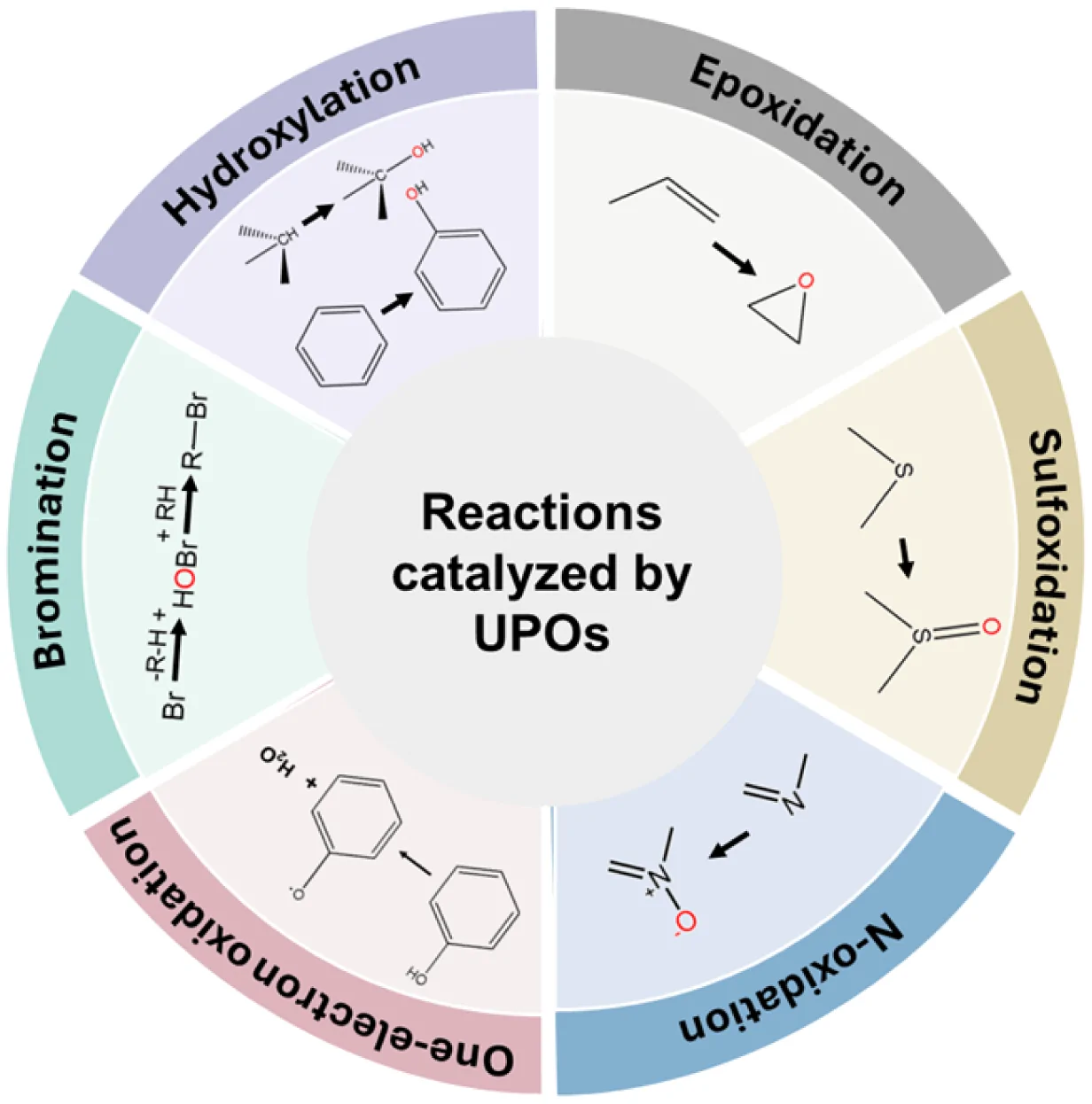
Selected reactions catalyzed by unspecific peroxygenases (UPOs).

## 3. Protein engineering strategies employed to enhance the properties of peroxygenases

Peroxygenases have emerged as highly attractive biocatalysts for pharmaceutical synthesis, enabling selective oxy-functionalization reactions that are difficult to achieve by traditional chemical methods. However, most naturally occurring peroxygenases lack the robustness, activity, and selectivity required for industrial application. Their practical application is limited by inefficient heterologous expression, susceptibility to H_2_O_2_-mediated inactivation, substrate-dependent selectivity, insufficient operational stability, and the difficulty of predicting how mutations will affect the heme environment and substrate-access architecture. Heterologous expression, particularly of fungal UPOs, remains a major bottleneck because production of catalytically active recombinant enzymes depends on efficient signal-peptide recognition and secretion, correct folding, heme incorporation, and appropriate post-translational processing. Differences between the native secretory machinery of the source organism and heterologous hosts can impair these processes and reduce the yield of correctly folded and catalytically active enzymes. Signal-peptide recognition and cleavage, glycosylation and host-specific folding machinery have been identified as important determinants of UPO production, while recent work has further shown that folding and heme incorporation can strongly influence the amount of active enzyme obtained in heterologous systems.^47,48^ Another major bottleneck is the dual role of H_2_O_2_ as both the required oxidant for UPO catalysis and a source of oxidative damage. Although H_2_O_2_ is essential for formation of the reactive heme intermediate, excess peroxide can promote peroxide-dependent inactivation involving compound III and hydroxyl-radical formation, ultimately leading to oxidative degradation of the heme porphyrin, heme bleaching, and loss of catalytic activity.^49^ UPO performance therefore depends on maintaining an appropriate balance between productive peroxide utilization and peroxide-induced oxidative degradation. Catalytic selectivity is closely linked to the architecture and dynamics of the active site. Substrate positioning relative to the reactive heme-oxo intermediate is governed by residues within the distal active site and access channel, such that even subtle structural changes can alter substrate orientation, accessibility, and regio- or chemoselectivity.^50–52^ Accordingly, reaction profiles can vary substantially depending on both the UPO scaffold and the substrate.^53^ Consequently, engineering outcomes can be difficult to predict, because mutations intended to improve substrate access or selectivity may simultaneously affect catalytic activity, stability, peroxide reactivity, or folding, creating trade-offs that complicate simultaneous optimization.

These challenges highlight the need for systematic protein engineering to tailor peroxygenases for pharmaceutical applications. Directed evolution and rational design have emerged as complementary strategies for improving heterologous expression, catalytic activity, H_2_O_2_ tolerance, operational stability, substrate scope, and regio-, chemo-, and stereoselectivity, as well as for reprogramming conventional P450 monooxygenases toward efficient H_2_O_2_-driven peroxygenase catalysis. More recently, AI-guided approaches have emerged as complementary tools for navigating the vast protein sequence space and for predicting, prioritizing, or generating promising enzyme variants, although their direct application to peroxygenase engineering remains at an early stage. Accordingly, this section discusses three major and increasingly interconnected strategies for peroxygenase engineering: directed evolution, rational and computationally aided design, and emerging AI-guided approaches.

### 3.1 Directed evolution

#### 3.1.1 P450 peroxygenase

Cytochrome P450s (CYPs) are optimal enzyme candidates for the biocatalytic synthesis of pharmaceuticals since these are involved in many biosynthetic pathways that produce natural and bioactive compounds in a wide range of organisms. Nowadays, novel P450 enzymes, reactions, and metabolites are continuously discovered, enabling the exploitation of their wide natural variability in pharmaceutical research. Moreover, protein engineering enables the generation of new P450 (CYP) variants with improved activity and high stereo-/regioselectivity for biocatalytic applications in the pharmaceutical field. For instance, Wong *et al.* performed directed evolution of P450BM3 for improved activity and conversion of hydrocarbon oxidation, producing human metabolites of a panel of drugs, such as cationic (amitriptyline and lidocaine), anionic (diclofenac and naproxen), and neutral (chlorzoxazone and testosterone) compounds.^54^

However, conventional P450 enzymes require expensive cofactors. To enhance their feasibility for large-scale or industrial synthesis, directed evolution has been applied to engineer cytochrome P450 into peroxygenases. In a pioneering research study, Arnold *et al.* employed directed evolution to engineer a *Pseudomonas putida* P450 enzyme, generating mutants capable of catalyzing hydroxylation *via* the ‘peroxide shunt’ pathway using H_2_O_2_ as a co-oxidant.^55^ To identify improved variants, a high-throughput screening strategy was implemented by co-expressing the P450 mutants with horseradish peroxidase, which transforms the P450's reaction products into fluorescent compounds detectable *via* digital imaging. This approach has yielded several variants with 20-fold improvements in naphthalene hydroxylation activity compared to the wild-type enzyme. It represents a groundbreaking work that marked the first successful demonstration of engineering P450 systems to function efficiently through the peroxide shunt mechanism, expanding the biocatalytic potential of these enzymes. Numerous efforts have thereafter been made to use directed evolution to transform P450 monooxygenases into peroxygenases. It is the case of cytochrome P450 (CYP 119) from *Sulfolobus acidocaldarius* that was engineered into a peroxygenase through directed evolution, marking the development of the first engineered CYP119 peroxygenase for styrene epoxidation with a high catalytic turnover frequency (308.8 min^−1^).^56^ Other than this, three other P450s (CYP199A4, CYP154C8, and CYP107) were modified into peroxygenases by altering the oxygen-binding region^57^ (discussed in Section 3.2.1.2).

Cytochrome P450s are essential in drug metabolism, with over 70% of drugs being processed in the liver by four main CYPs (CYP3A4, CYP2C9, CYP2D6, and CYP1A2) at some stage in their metabolic pathway. However, producing these metabolites *in vitro* using human cytochrome P450 enzymes is difficult due to their high cost, instability, and low activity. Arnold *et al.* conducted directed evolution on cytochrome P450 BM3 from *Bacillus megaterium* (CYP102A1). Through rounds of random mutagenesis and high-throughput screening, P450 BM3 heme domain peroxygenase (BM3-H) was identified, which efficiently hydroxylates fatty acids using hydrogen peroxide instead of NADH/O_2_, acting as a self-sufficient peroxy-driven hydroxylation biocatalyst.^58^ With further evolution, variants of P450 BM3-H were found that could convert propranolol into primary human metabolites, especially producing higher amounts of key ring-hydroxylated metabolites such as 4′-hydroxypropranolol (4′-OHP) and 5′-hydroxypropranolol (5′-OHP).^59^ This offers a versatile, cost-effective, and scalable method for synthesizing drug metabolites.

Atorvastatin is a widely used cholesterol-lowering drug in treating hypercholesterolemia and preventing cardiovascular disease. The atorvastatin can be hydroxylated to 2-OH and 4-OH atorvastatin by cytochrome P450 (CYP3A4) in the human liver. Yun *et al.* examined the peroxygenase activity of CYP102A1 towards atorvastatin. After a directed evolution campaign, a CYP102A1 peroxygenase variant, with nine changed amino acid residues, was identified showing the highest catalytic turnover for atorvastatin 4-hydroxylation (1.8 min^−1^).^20^ This result highlights CYP102A1 competence to catalyze atorvastatin 4-hydroxylation by peroxide-dependent oxidation with high catalytic activity.

Hydroxyl groups are common functional groups found in many approved drugs, playing a crucial role in shaping compounds' pharmacokinetic and physicochemical properties. Despite their significance, the selective introduction of hydroxyl groups onto unreactive C–H bonds poses a major challenge in synthetic chemistry. Cytochrome P450s have been used for the hydroxyl functionalization of inactivated C–H bonds and elucidated in numerous studies. For example, Schwaneberg *et al.* reported the P450BM3 variant M2 for the aromatic hydroxylation of halogenated benzenes, toluene, and anisole. These relative phenol products are important building blocks in the syntheses of FDA-approved drugs, including liothyronine, atomoxetine, and vancomycin.^60^ In this review, we focus on the directed evolution of P450 peroxygenases for C–H hydroxylation of fatty acids and derivatives, targeted for synthetic and pharmacological studies. Chen *et al.* employed directed evolution strategies for iterative mutagenesis of P450BSβ, resulting in a quadruple mutation variant that enables highly regio- and enantioselective β-hydroxylation of various fatty acid substrates, *e.g.*, myristic acid, with excellent enantioselectivity (>99% ee, total turnover numbers (TTNs) up to 1540).^61^ Similarly, Wang *et al.* performed directed evolution for P450BSβ, realizing β-hydroxylation of inactivated C–H bonds in a broad spectrum of aliphatic carboxylic acids with high chemo-, regio-, and enantioselectivity (>30 examples, C_β_/C_α_ > 20 : 1, >99% ee).^62^ Based on this study, the directed evolution of P450BSβ for enantioselective γ-lactonization of inactivated C(sp^3^)–H bonds of aliphatic carboxylic acids was repurposed. γ-Lactones have broad applications in the food, cosmetic, and pharmaceutical industries. The generated variant P450BSβ-L78G/Q85F/F173S/G290I successfully executed a direct oxidative γ-lactonization of aliphatic carboxylic acids with high efficiency (TTN 3278) in an enantioselective manner, an extremely difficult reaction with small-molecule catalysts.^63^

#### 3.1.2 Unspecific peroxygenases

The synthesis of human drug metabolites (HDMs) by UPOs is a topic of great interest for the pharmaceutical industry. By emulating the function of human liver cytochrome P450 enzymes, UPOs provide a wide variety of reactions for pharmaceutical studies. Despite their great potential, their practical application in the industry remains limited due to generally low homologous or heterologous production and insufficient robustness under operational conditions. To date, less than 1% of the identified putative UPO sequences have been fully characterized, with only 22 UPO enzymes successfully expressed functionally in either their native fungal hosts or through heterologous systems.^64^

Heterologous expression is essential for peroxygenase engineering and industrial applications as it enables the modification of enzyme properties through directed evolution. The first successful heterologous expression of a UPO enzyme was reported in 2014 in yeast systems, including *Saccharomyces cerevisiae* and *Pichia pastoris*.^65,66^ Through directed evolution of the *Agrocybe aegerita* UPO (*Aae*UPO) gene, a variant named PaDa-I was generated by introducing four mutations in the signal peptide and five mutations in the mature enzyme (F12Y, A14V, R15G, A21D, V57A, L67F, V75I, I248V, and F311L). This engineered variant demonstrated a significant increase in expression and catalytic activity, reaching production levels of 8 mg L^−1^ in *Saccharomyces cerevisiae* and 217 mg L^−1^ in *Pichia pastoris*. These improvements in heterologous expression represent an important milestone toward industrial implementation as enzyme production cost remains one of the major contributors to overall process economics.^66,67^ However, expression titres reported for many engineered UPOs remain highly variable and, in many cases, are still below those typically required for economically viable large-scale biocatalytic manufacturing.^67,68^ Consequently, further optimization of expression hosts, secretion pathways, and fermentation processes remains an important objective for the industrial translation of engineered peroxygenases.

Building on this progress, several other fungal UPOs, such as those from *Marasmius fiardii* PR910 and *Galerina marginata*, were successfully expressed in yeast systems.^67,69^ More recently, progress has also been made in prokaryotic hosts: soluble expression of UPO from the ascomycetes *Collariella virescens* and *Daldinia caldariorum* has been achieved in *Escherichia coli* at “mg-per-liter” levels.^68^ More recently, computational enzyme mining has begun to reshape the UPO discovery. A preprint study by Shen *et al.* applied a deep-learning-based enzyme mining strategy to uncover six bacterial UPOs: *Myx*1UPO, *Myx*2UPO, *Pse*UPO, *Del1*UPO, *Del2*UPO, and *Pol*UPO. All six enzymes exhibited robust heterologous expression in *E. coli*, with production titers of 28.2–35.0 mg L^−1^.^70^ Furthermore, structure-database mining has further discovered a new family of bacterial UPO-like enzymes (BUPO), structurally analogous to short UPOs. Three representative BUPOs, *Age*BUPO from *Archangium gephyra*, *Kal*BUPO from *Ketobacter alkanivorans*, and *Hyd*BUPO from *Hydrogenophaga* sp. A37, were successfully expressed in *E. coli*, yielding approximately 40, 10, and 10 mg L^−1^ of BUPO production, respectively. These recent advances highlight the potential of computational and structure-guided mining strategies to expand the repertoire of bacterial UPOs amenable to heterologous production in *E. coli*.^71^Table 4 provides the current heterologous expression of UPOs in *Escherichia coli* and yeast systems.

**Table 4:** The current heterologous expression systems of UPOs

Enzyme	Source organism	Family	Expression organism	Expression yield (mg L^−1^)	References
*rAae* UPO (PaDa-I variant)	*Agrocybe aegerita*	Long	*S. cerevisiae*	8.0	66
			*P. pastoris*	217.0	
*rCgl*UPO	*Chaetomium globosum*	Short	*S. cerevisiae*	0.6	67
			*P. pastoris*	9.0	
*rMfe*UPO	*Myceliophthora fergusii*	Short	*P. pastoris*	6.5	72
*rMhi*UPO	*Myceliophthora hinnulea*	Short	*Pichia pastoris*	5.7	72
*rMth*UPO	*Myceliophthora thermophila*	Short	*S. cerevisiae*	5.0	67
			*P. pastoris*	24.0	
*rMwe*UPO	*Marasmius wettsteinii*	Short	*S. cerevisiae*	700.0	73
*rCvi*UPO	*Chaetomium virescens*	Short	*E. coli*	19.7	68
*rDca*UPO	*Daldinia caldariorum*	Short	*E. coli*	2.8	68
*rMro*UPO	*Marasmius rotula*	Short	*S. cerevisiae*	0.4	67
			*P. pastoris*	1.1	
*rTte*UPO	*Thielavia terrestris*	Short	*S. cerevisiae*	17.0	67
			*P. pastoris*	14.0	
*rMfi*UPO	*Marasmius fiardii*	Short	*P. pastoris*	1180.0	69
*Age*BUPO	*Archangium gephyra*	Analogous to short	*E. coli*	40.0	71
*Kal*BUPO	*Ketobacter alkanivorans*	Analogous to short	*E. coli*	10.0	71
*Hyd*BUPO	*Hydrogenophaga* sp. A37	Analogous to short	*E. coli*	10.0	71
*Myx*1UPO	Bacterial derived	—	*E. coli*	28.2	70
*Myx*2UPO	Bacterial derived	—	*E. coli*	30.0	70
*Pse*UPO	Bacterial derived	—	*E. coli*	34.0	70
*Del*1UPO	Bacterial derived	—	*E. coli*	32.0	70
*Del2*UPO	Bacterial derived	—	*E. coli*	33.0	70
*Pol*UPO	Bacterial derived	—	*E. coli*	35.0	70

Additionally, the directed evolution campaign highly relies on precise screening systems that can identify beneficial variants from a mutagenesis library. Based on the wide substrate scope of UPOs (*e.g.*, more than 400 substrates are now known) that cover several different reaction types, some colorimetric-based high-throughput screening (HTS) assays are now available for mutant library screening. The colorimetric HTS assays can be grouped into the peroxidase activity-based and the peroxygenase activity-based. For the peroxidase activity-based HTS assay, the oxidation of 2,2-azino-bis-3-ethylbenzothiazoline-6-sulfonic acid (ABTS) and 2,6-dimethoxyphenol (DMP) is the typical reaction for screening UPO peroxidase activity. For the peroxygenase activity-based HTS assay, 5-nitro-1,3-benzodioxole (NBD) and naphthalene (Fast Red) are the typical reactions for screening UPOs' activity.^47^ The colorimetric-based HTS assays are summarized in Table 5.

**Table 5:** High throughput screening assays for the directed evolution of UPOs

Substrate	Substrate	Reaction	Product	Readout (*λ*_max_)	CV	References
ABTS	2,2′-Azino-bis(3-ethylbenzothiazoline-6-sulfonic acid)	One-electron oxidation	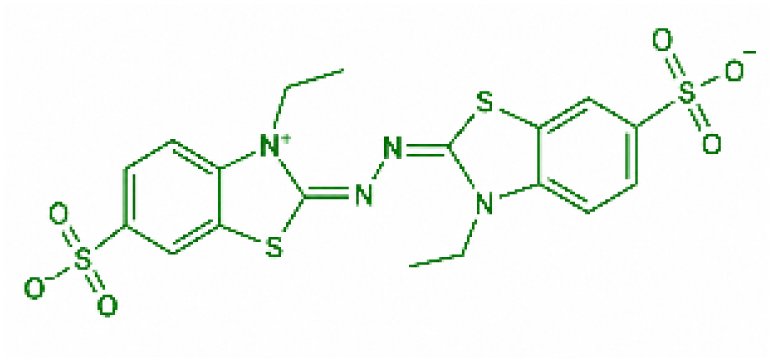	418 nm	<12%	65
DMP	2,6-Dimethoxyphenol	One-electron oxidation	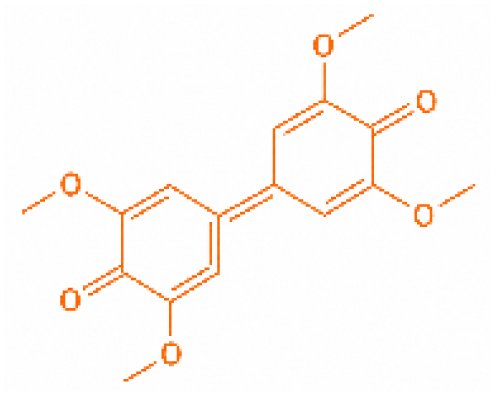	469 nm	<12%	72 and 74
*o*-Dianisidine	3,3′-Dimethoxybenzidine	One-electron oxidation	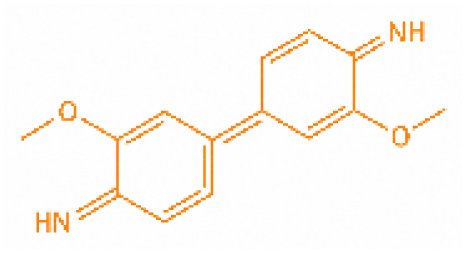	460/540 nm	19%	75
Amplex Red	10-Acetyl-3,7-dihydroxyphenoxazine	One-electron oxidation	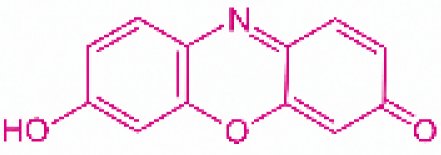	563/540–590 nm	17%	76
TMB	3,3′,5,5′-Tetramethylbenzidine	One-electron oxidation	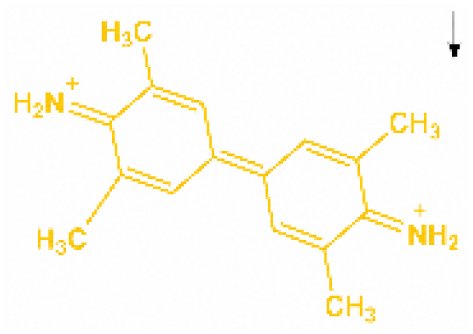	450/653 nm	9%	77
Purpald	4-Amino-3-hydrazino-5-mercapto-1,2,4-triazole	*O*-Dealkylation, *N*-dealkylation	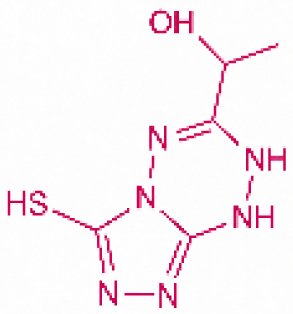	500–550 nm	19%	24
4-AAP	4-Aminoantipyrine	Aromatic hydroxylation	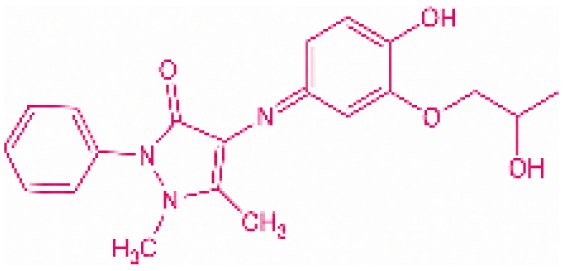	510 nm	12%	15
NBD	7-Nitro-1,3-benzodioxole	*O*-Dealkylation	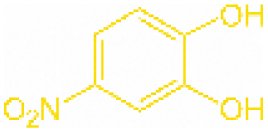	425 nm	<12%	72
NBP	4-(4-Nitrobenzyl)pyridine	Styrene epoxidation	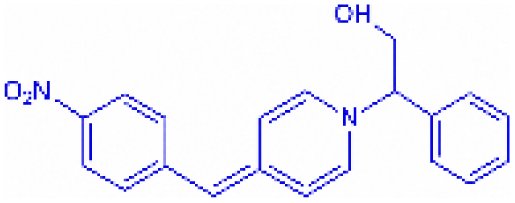	600 nm	11%	78
Russig's blue	Oxidation product of indole	Aromatic hydroxylation	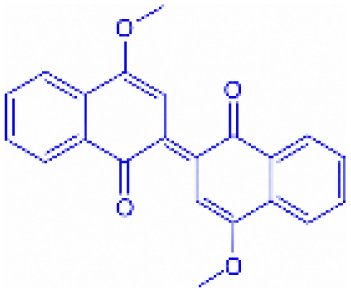	635 nm	14%	79
Indole	Indole	Heterocycle hydroxylation	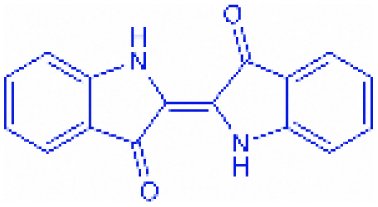	670 nm	17%	80
Fast Red	Fast red TR diazonium salt	Naphthalene hydroxylation	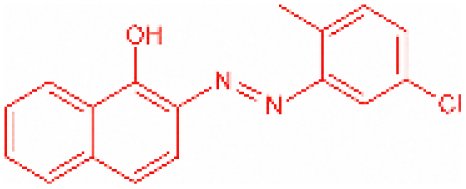	510 nm	<12%	81

By mimicking human liver cytochrome P450s, the HDMs synthesized by UPOs have been comprehensively researched recently. It has been proven that by coupling with oxidases, UPOs can convert 33 out of 64 drugs. For example, Scheibner *et al.* used *Mro*UPO for the hydroxylation of cyclophosphamide (CPA, a widely used anti-cancer drug), to produce HDM 4-hydroxycyclophosphamide (4-OH-CPA) with a yield of 32% (>97.6% purity, TTN 10 000).^82^ Oxyfunctionalization of testosterone (a bulky steroid) by UPO from *Chaetomium globosum* (*Cgl*UPO) was also investigated, achieving the formation of 90% of the 4,5-epoxide product and 10% HDM 16α-hydroxytestosterone, with high turnover (TTN 7000) and purity (>98%).^25^ With the potential of HDM synthesis by UPOs, Alcalde *et al.* performed the directed evolution of *Aae*UPO in *Saccharomyces cerevisiae* for the highly efficient and selective synthesis of 5′-hydroxypropranolol (5′-OHP) from propranolol. With the sensitive 4-AAP screening assay, an *Aae*UPO variant SoLo (Jawa_F191S) was demonstrated to significantly enhance catalytic efficiency for the synthesis of 5′-hydroxypropranolol (5′-OHP) from propranolol. Compared with the wild-type enzyme, this variant achieved a 15-fold increase in regioselective transformation, producing >99% 5′-OHP from propranolol.^15,16^ And when combining the reaction with an *in situ* H_2_O_2_ generation system, the SoLo mutant achieved TTNs up to 420 000. Evolved *Aae*UPO variants have also been applied to the synthesis of additional drug metabolites. For instance, the SoLo variant converted dextromethorphan to dextrorphan by *O*-dealkylation with a reported TTN of 62 000 and a yield of 75.2%.^24^*Aae*UPO variants were also reported for the demethylation of naproxen and the hydroxylation of tolbutamide, validating the promising prospect for synthesizing HDMs by UPOs.^83^

Another relevant example of a UPO's application for HDMs is in vitamin biotransformations. Vitamin D (calciferols) is a group of lipophilic steroids, which are essential for calcium and phosphate homeostasis in animal metabolism. The 25-hydroxylated metabolites of vitamin D have been proven to be better therapeutic agents for several diseases than vitamin D itself. Gutiérrez *et al.* reported that the UPO from *Coprinopsis cinerea* can be used for complete regioselective hydroxylation of vitamin D3 (cholecalciferol) and vitamin D2 (ergocalciferol) at the C-25 position, with the production of 25-hydroxycalciferol.^84^ More recently, Liu *et al.* performed computational-aided directed evolution for the evolution of *Aae*UPO for efficient and regioselective hydroxylation of vitamin Ds at the C-25 position. The best two variants, *Aae*UPO_Stev and *Aae*UPO_Veco, showed a 25- to 27-fold increase in enzyme activity with an improvement in selectivity from 25% to over 93%, enabling the conversion of vitamin D3 to 25-hydroxyvitamin D3 on a gram scale.^85^

Vitamin K3 (menadione) and the vitamin K family comprise 2-methyl-1,4-naphthoquinone derivatives. In another research study, *Mth*UPO was engineered in *Saccharomyces cerevisiae* through structure-guided directed evolution for chemo-/regioselective oxidation of naphthalene derivatives. After an extensive screening of more than 5300 variants *via* an NBD based high-throughput assay, the variant L60F/S159G/A161F was identified, which oxidizes 2-methylnaphthalene at the substituted ring and shifts the major product formation to 2-methyl-1,4-naphthoquinone (vitamin K3), demonstrating the regioselectivity of this variant.^85^ The engineered UPO selectivity provides a one-step direct route to access these vitamin K molecules.

Peroxygenases selectively incorporate oxygen into organic molecules, paving a ‘green’ route for pharmaceutical synthesis, as many of their transformation products are the fundamental intermediates of pharmaceuticals. Sigmund *et al.* enhanced the peroxygenase activity of 4-oxalocrotonate tautomerase (4-OT) through directed evolution. With five rounds of iterative evolution and screening, a tandem-fused 4-OT variant was identified and realized 150-fold enhanced activity in catalysing the epoxidation of cinnamaldehyde, enabling the synthesis of epoxides (versatile synthons for production of pharmaceuticals) in mg- and g-scale with high enantiopurity (up to 98% ee) and excellent conversion.^86^ Moreover, Weissenborn *et al.* performed the directed evolution of *Mth*UPO, the identified variants achieve high chemo- and regioselectivities in the oxyfunctionalization of aromatic and benzylic carbons, respectively. The benzylic hydroxylation was demonstrated to perform with enantioselectivities of up to 95% ee and TTN up to 8160. The proposed evolutionary protocol represents a way toward the broader application of UPOs in organic chemistry and the pharmaceutical industry.^87^

### 3.2 Rational design

#### 3.2.1 P450 peroxygenase

Rational design strategies have been applied to improve the performance of natural peroxide-dependent enzymes and to convert canonical cytochrome P450 monooxygenases into H_2_O_2_-driven peroxygenases. Engineered P450 peroxygenases are highly relevant for pharmaceutical biocatalysis as they offer a simpler catalytic system for selective hydroxylation, epoxidation, *O*-demethylation, and heteroatom oxidation reactions relevant to drug metabolite synthesis, steroid derivatization, and late-stage functionalization of bioactive molecules.

Regioselective α-hydroxylation of fatty acids is an important catalytic reaction mediated by peroxygenases, yet its practical application is hindered by poor stability in the presence of H_2_O_2_. Kroutil *et al.* employed the Protein Repair One Stop Shop (PROSS)^88^ strategy to design a minimal enzyme library to simultaneously improve multiple properties, including H_2_O_2_ tolerance, specific activity, and expression yield.^89^ Through this approach, they identified the CYP152 peroxygenase POSPα with up to a 4-fold enhancement in hydrogen peroxide tolerance. Among 12 generated variants, the variant V3-P04 exhibited the most significant overall improvements, outperforming the parent enzyme in H_2_O_2_ tolerance, expression yield, and specific activity toward the α-hydroxylation of octanoic acid. This study shows that computer-aided scaffold stabilization can improve the functional robustness of naturally peroxide-dependent P450 enzymes. In parallel with the optimization of natural P450 peroxygenases, rational design has been extensively applied to reprogram canonical P450s. Conversion of P450 monooxygenases into peroxygenases *via* rational design has mainly focused on three complementary strategies: engineering the oxygen-binding groove to improve H_2_O_2_ activation, introducing exogenous acid–base catalysts through dual-functional small molecules, and improving peroxide delivery through H_2_O_2_-access tunnel engineering.^90^

##### 3.2.1.1 Oxygen-binding groove engineering

A central design principle involves reprogramming of the oxygen activation machinery to favor hydrogen peroxide–driven catalysis. In native P450s, the I-helix region above the heme contains a conserved acid–alcohol pair (typically Asp/Glu–Thr) that mediates proton delivery during O_2_ activation.^57,91^ Rational engineering of this region often involves replacing the conserved threonine with an acidic residue such as aspartate or glutamate, thereby introducing an acid–base catalytic functionality within the oxygen-binding groove. This modification promotes the heterolytic cleavage of H_2_O_2_ and direct formation of the reactive compound I intermediate, enabling regio- and stereoselective oxidation reactions relevant to pharmaceutical synthesis.^57,91^

This design strategy has been successfully applied to several P450 enzymes. For example, mutation of the conserved threonine residue in CYP199A4 from *Rhodopseudomonas palustris* HaA2 (T252E) introduced an acid–base catalytic residue that enhanced peroxygenase activity towards *para*-substituted benzoic acids, catalyzing reactions such as *O*-demethylation of 4-methoxybenzoic acid to 4-hydroxybenzoic acid, veratric acid to vanillic acid, and the enantioselective epoxidation of 4-vinylbenzoic acid, producing the (*S*)-epoxide with up to ∼92% ee.^57,92–94^ Further optimization generated the QE double mutant (D251Q/T252E), designed to mimic the glutamine–glutamate catalytic motif observed in natural peroxygenases, which improved the H_2_O_2_-driven activity relative to both the T252E mutant and the wild-type enzyme.^57^

A similar strategy was applied to the thermostable cytochrome P450 CYP119 from *Sulfolobus acidocaldarius*, where seven residues of the I-helix (AGNETTT) were replaced with GALQEPG, generating the variant CYP119-perox7.^91^ This engineered variant showed enhanced fatty-acid hydroxylation, with over 10-fold higher activity at room temperature and up to 35-fold higher activity at 80 °C compared with the wild-type enzyme.

Comparable engineering has also been demonstrated in the steroid-hydroxylating enzyme CYP154C8 from a *Streptomyces* bacterium.^95^ Replacement of the conserved I-helix threonine (T258E) generated a peroxygenase capable of regio- and stereo-selective hydroxylation of steroids such as progesterone and androstenedione, exhibiting over 100-fold higher peroxygenase activity than the wild-type enzyme. In another example, the modification of the I-helix of CYP107PQ1 from *Meiothermus ruber* generated the peroxygenase variant CYP107PQ1QE (E247Q/A248E), capable of the enantioselective oxidation of β-ionone to (*S*)-4-hydroxy-β-ionone using H_2_O_2_ as the oxidant (∼94% ee).^96^ Collectively, these studies demonstrate that targeted modification of the oxygen-binding groove represents an effective rational design strategy for converting monooxygenases into peroxygenases, enabling efficient peroxide-driven catalysis while maintaining high regio- and stereoselectivity toward diverse substrates.

##### 3.2.1.2 Dual-functional small molecule (DFSM) assisted catalysis

An alternative strategy for converting cytochrome P450 monooxygenases into efficient peroxygenases involves the use of dual-functional small molecules (DFSMs). In contrast to the direct protein engineering approach of the above-mentioned oxygen-binding groove, this approach introduces an external small molecule that simultaneously anchors within the active site and provides an acid–base catalytic group to facilitate hydrogen peroxide activation.^96^ DFSMs consist of two key functional components: (i) an anchoring acyl amino acid moiety that binds within the enzyme's substrate channel and (ii) a distal basic group (typically imidazole) that facilitates heterolytic cleavage of H_2_O_2_. This rational design mimics the catalytic properties of natural peroxygenases and promotes efficient formation of the high-valent compound I intermediate through heterolytic O–O bond cleavage.^96^

The most extensively used DFSM platform consists of P450BM3 variants assisted by *N*-(ω-imidazolyl)-hexanoyl-l-phenylalanine (Im-C6-Phe). Im-C6-Phe was first used to activate normally H_2_O_2_-inert P450BM3 variants, enhancing peroxygenase activity without NAD(P)H or redox partner proteins. In the presence of Im-C6-Phe, P450BM3 variants achieved a maximum TTNs of 2733, 4910, and 730 for styrene epoxidation, thioanisole sulfoxidation, and ethylbenzene hydroxylation, respectively.^97^ The same Im-C6-Phe-assisted P450BM3 platform was later extended to the peroxide-driven hydroxylation of small alkanes.^98^ This system exhibited TTNs comparable to the fungal peroxygenase *Aae*UPO (the only known natural H_2_O_2_-dependent alkane hydroxylase) and achieved similar or higher product formation rates than engineered NADPH-dependent P450s.^98^

In addition to simple model reactions, the Im-C6-Phe/P450BM3 system has been applied to more synthetically relevant transformations. P450BM3 variants combined with Im-C6-Phe were also used for the H_2_O_2_-driven synthesis of indigoid dyes from indoles and their derivatives.^99^ The variant F87G/T268V/F77I/E140D selectively catalyzed C3-hydroxylation of indole followed by spontaneous oxidation to indigo, reaching a TTN of 6588 and 65.8% isolated yield on a preparative scale. The system was further extended to substituted indoles, producing 12 indigoid dyes in 31.3–79.5% isolated yields. Similarly, Im-C6-Phe-assisted P450BM3 variants were used for divergent *N*-oxidation of primary aromatic amines.^85^ The reaction pathway could be switched between two-electron *N*-oxygenation and one-electron oxidation: F87A/T268V/V78T/A82T with Im-C6-Phe selectively converted *p*-toluidine to *p*-nitrosotoluene (up to 98% selectivity), whereas F87A/T268V/A82T/I263L with Im-C6-Tyr-Nap produced *p*-nitrotoluene with >99% selectivity. In contrast, the bulky F87L/V78S/A184V variant favored one-electron oxidation, generating azo and oligomeric products. This study shows that DFSMs not only activate H_2_O_2_ but also cooperate with active-site mutations to control substrate orientation and switch P450BM3 between peroxygenase- and peroxidase-like *N*-oxidation pathways.

The DFSM strategy was further expanded by modifying the structure of the small molecule itself and by coupling the system with auxiliary peroxide-generation enzymes. The combination of engineered P450BM3 variants with different DFSMs such as *N*-(ω-imidazolyl)-hexanoyl-l-phenylalanine (Im-C6-Phe) and *N*-(ω-imidazolyl)pentanoyl-l-phenylalanine (Im-C5-Phe), enabled regiodivergent and enantioselective H_2_O_2_-driven hydroxylation of alkylbenzenes.^100^ Substrates, including toluene, ethylbenzene, propylbenzene, indane, and tetralin, were selectively converted into distinct benzylic alcohols and phenolic products, with regioselectivity up to >99%, enantioselectivity up to >99% ee, and TTN up to 80 963 using different P450BM3 variant-DFSM combinations. These results showed that DFSM can both promote peroxide activation and influence substrate positioning, demonstrating the potential of exogenous small molecules on catalytic activity and regio-/stereoselectivity. The replacement of the imidazole catalytic group with pyridyl groups in the DFSM-assisted P450BM3 peroxygenase further improved its efficacy.^101^ The resulting Pyd-DFSMs markedly enhanced C(sp^3^)–H hydroxylation of medium-chain alkanes and cycloalkanes (up to 12-fold higher TTN for heptane and 17-fold higher TTN for cycloheptane). It also modulated regio- and enantioselectivity, including altered stereoselectivity in benzylic hydroxylation and improved allylic hydroxylation selectivity of terminal alkenes. Xu *et al.* combined engineered P450BM3 variants with AldO (sugar alcohol oxidase) and DFSM (Im-C6-Phe) to develop a peroxygenase system for regioselective *O*-demethylation of lignin-derived aromatic ethers, in which AldO generated H_2_O_2_*in situ* from glycerol to avoid peroxide overaccumulation and enzyme inactivation.^102^ The variant F87A/V78A/T268D/A328F converted guaiacol to catechol with a TTN of 1895 ± 4 and 99.9% selectivity, more than double that of the native P450BM3/H_2_O_2_ system, while F87A/T268D catalyzed double demethylation of syringol to pyrogallol with a TTN of 483 ± 7 and 92% selectivity.

Collectively, these studies demonstrate that DFSMs provide an efficient modular strategy to reprogram P450BM3 into H_2_O_2_-driven oxidation catalysts. The enzyme scaffold, DFSM structure, and peroxide-delivery mode can each be tuned to control catalytic activity, substrate scope, and regio-/stereoselectivity.

##### 3.2.1.3 H_2_O_2_ tunnel engineering

Another rational design strategy for converting P450 monooxygenases into peroxygenases is H_2_O_2_ tunnel engineering (HTE). Since the heme center of P450s is deeply buried within the protein scaffold, poor H_2_O_2_ accessibility can limit peroxide-shunt catalysis. HTE addresses this by remodeling native water channels that connect the active site to the bulk solvent, taking advantage of the physicochemical similarity between water and H_2_O_2_. Cong *et al.* first demonstrated this strategy using CAVER-guided substitutions of water-tunnel residues to convert NADH-dependent P450s into H_2_O_2_-driven peroxygenases.^103^ Mutation of tunnel bottleneck residues in CYP199A4 from *Rhodopseudomonas palustris* HaA2, enabled H_2_O_2_-driven *O*-demethylation of 4-methoxybenzoic acid to 4-hydroxybenzoic acid with up to >183-fold improvement over the wild type.^103^ Crystallographic studies and molecular dynamics simulations identified the crucial gating amino acids in CYP199A4 (F182 and D251). Replacement of bulky F182 with alanine opened the H_2_O/H_2_O_2_ tunnel and D251 organized the hydrogen-bond.^104^ Engineered CYP199A4 variants further improved the *O*-demethylation of diverse methoxy-substituted benzoic acids, giving TTNs above 1000 for selected substrates.^104^ The HTE strategy applied to CYP153A_M.aq_ from *Marinobacter aquaeolei* improved H_2_O_2_-driven hydroxylation of dodecanoic acid to 12-hydroxydodecanoic acid by up to 15-fold.^103^ A similar strategy was employed to engineer CYP105D18 from *Streptomyces laurentii* into an H_2_O_2_-dependent steroid dihydroxylase.^105^ Tunnel engineering guided by integrated molecular dynamics simulation and crystallography analysis resulted in a variant (F184A/F191A/E196A) with an approximately 80-fold increase in catalytic efficiency for testosterone hydroxylation.

Tunnel engineering has also been combined with other rational engineering approaches. For instance, CYP154C5 from *Nocardia farcinica* was converted into steroid-hydroxylating peroxygenases by integrating HTE with the oxygen-binding groove.^106^ Specifically, tunnel mutations with replacement of the conserved I-helix threonine by aspartate, resulted in an enzyme variant (F92A/R114A/E282A/T248D) catalyzing selective 16α-hydroxylation of testosterone and nandrolone. The optimized enzyme achieved a >98% substrate conversion rate, a TTN close to 953, and a 90% isolated yield of 16α-hydroxytestosterone on a mg scale.^106^ HTE also enhanced DFSM-facilitated artificial P450 peroxygenases, such as the Im-C6-Phe/P450BM3 system.^103^ Screening tunnel-entrance residues in the GV variant (F87G/T268V) identified D182G and L272S, which improved peroxide-driven indole hydroxylation, styrene epoxidation, ethylbenzene hydroxylation, and thioanisole sulfoxidation by up to 258%.^103^ Notably, GV/L272S reduced the required H_2_O_2_ concentration by 95–97.5% while exhibiting ∼95% coupling efficiency.

Overall, HTE provides a general rational design strategy for converting P450 monooxygenases into peroxygenases, either alone or in combination with complementary approaches. By improving peroxide access to the buried heme center, HTE enables efficient peroxygenase catalysis for pharmaceutically relevant transformations, including steroid hydroxylation, aromatic *O*-demethylation, and selective C–H oxyfunctionalization.

#### 3.2.2 Unspecific peroxygenases (UPOs)

Unspecific peroxygenases (UPOs) exhibit excellent regio-, chemo- and stereoselectivity under environmentally friendly conditions, underscoring their strong potential as catalysts for selective C–H oxyfunctionalization in pharmaceutical synthesis. However, the practical application of native UPOs is often limited by low activity, poor heterologous expression, regio- or enantioselectivity and oxidative inactivation by H_2_O_2_. While directed evolution has proven to be a powerful strategy for enhancing enzyme performance, the detection of oxyfunctionalization products is often not amenable to chromogenic or fluorogenic assays, limiting the development of medium- to high-throughput screening methods. Computationally aided rational design provides an efficient complementary strategy by enabling identification of functional hotspots and construction of smart or focused libraries, thereby accelerating the engineering of key enzyme properties. Rational design has been used to improve heterologous expression, enzyme activity, stability, substrate access, and regio-/enantioselectivity, as summarized by examples below.

In a recent study, Wu *et al.* combined five different computational approaches, including HotSpot Wizard, molecular docking, substrate-channel analysis, multiple sequence alignment, and molecular dynamics (MD) simulations, to identify hotspot residues for *Collariella virescens* UPO (*Cvi*UPO) engineering.^107^ Screening a rationally designed focused library, they identified top-performing *Cvi*UPO variants that exhibited up to 13-fold increased activity and >99% enantioselectivity for C–H oxidation. More recently, a study by Gao *et al.* addressed the stability–activity–selectivity trade-off in *Cvi*UPO using FoldX-guided global mutational analysis, spatial residue prioritization, and MD simulations. The resulting variant M1_T9P_G161L showed a 36-fold longer half-life and 6-fold higher activity than the parental M1 variant while retaining >99% ee, corresponding to a 75-fold activity improvement relative to wild type.^108^

Li *et al.* combined ancestral sequence reconstruction (ASR) and substrate-binding pocket engineering for the rational design of UPOs for steroid hydroxylation, enabling the production of valuable intermediates for the synthesis of a wide range of steroid-based pharmaceuticals.^109^ They obtained the ancestral UPO N1 using ASR, which exhibited 3-fold improved expression with 63% regioselectivity toward steroid hydroxylation. Subsequently, MD simulations were employed to guide the rational mutagenesis of key substrate-binding residues. From the resulting mutagenesis library, the best-performing variant was identified with a 36-fold enhancement in catalytic activity and near-complete 11β-regioselectivity (99%). This variant also displayed diverse hydroxylation patterns (11β-, 16α-, and 6β-), enabling gram-scale synthesis of various hydroxylated steroids with 40–86% yield.

Ionones, also known as rose ketones, are important building blocks for the synthesis of carotenoids and retinol (vitamin A). Their hydroxylated derivatives exhibit diverse biological activities, including anticancer, chemopreventive, melanogenesis-promoting, anti-inflammatory, and antimicrobial effects. Weissenborn *et al.* sought to enhance the enantioselectivity of short-type *Mth*UPO toward ionones.^110^ They employed a computational strategy based on substrate-bound restrained MD simulations to investigate near-attack conformations involved in selective hydroxylation and to characterize the relevant binding modes of the model substrate β-ionone. Guided by these insights, they designed a focused smart library and screened the variants using a GC-MS-based assay. After two iterative rounds of protein engineering, the best variant exhibited up to a 17-fold increase in activity and achieved regioselectivity as high as 99.6% for the production of 4-hydroxy-β-ionone.

Similarly, Zheng *et al.* developed a molecular coevolution approach, termed the substrate-binding pocket co-evolution strategy (SBPCS), which targets key functional residues within the enzyme's substrate-binding pocket to achieve regioselective hydroxylation of vitamin D3 by *Agrocybe aegerita* UPO (*Aae*UPO).^111^ By combining mutational screening of key residues within the substrate-binding pocket with MD simulations, they identified variants that shortened the distance and stabilized the dihedral angle between the substrate C25 atom and the heme Fe=O catalytic center. Following two rounds of coevolution, a triple variant M3 (G195A/V244I/S272A) was obtained, exhibiting a 5.16-fold improvement in C25-hydroxylation efficiency and increasing the yield of 25(OH)VD_3_ from 45.8 to 354.40 mg L^−1^.

The FuncLib^112^ algorithm, which integrates phylogenetic analysis with Rosetta-based design calculations to generate multipoint enzyme active sites mutations, has also been applied for UPO engineering. Alcalde *et al.* employed FuncLib to engineer *Aae*UPO for improved enantioselective C–H oxyfunctionalization.^113^ In a single-round of computational design, they generated 24 variants, each containing four to five mutations within the catalytic core. Several of these variants displayed remarkable enantiodivergence and up to a 15-fold increase in TTNs relative to the wild-type enzyme. Similarly, Weissenborn *et al.* applied FuncLib to engineer *Mth*UPO for the selective oxyfunctionalization of terpenes, a class of compounds whose oxygenated derivatives frequently possess valuable pharmacological properties.^114^ Using the algorithm, they constructed a focused library of only 50 variants, many of which showed substantial functional enhancements across a panel of terpene substrates. Depending on the substrate tested, the top-performing variants showed activity improvements ranging from 2.2-fold to 7.1-fold compared with the wild type. In addition to enhanced catalytic activity, numerous variants exhibited improved regio-, chemo-, and stereoselectivity. For instance, regioselectivity for the previously less-favoured 3-hydroxy-β-damascone increased from 3% to 46%. Likewise, chemoselective oxidation of geraniol and nerol to citral A and citral B was significantly improved (citral A: from 40% to >99%; citral B: from 72% to 89%), respectively, both of which are important intermediates in vitamin synthesis. These studies demonstrated the potential of *silico*-guided approaches to rapidly develop highly selective biocatalysts for synthetic applications.

Beyond direct sequence engineering, UPO performance can also be improved through enzyme-fusion strategies that address peroxide delivery. For example, the SoLo variant of *Aae*UPO was fused to the evolved aryl-alcohol oxidase variant (FX9) to construct a self-sufficient UPO–AAO catalyst.^24^ The resulting bifunctional enzyme generated H_2_O_2_*in situ* during aromatic alcohol oxidation, eliminating the need for external H_2_O_2_ addition and enabling dextrorphan synthesis with a TTN of up to 62 000.

Collectively, these studies demonstrate that rational design provides a powerful route for improving activity, selectivity, and stability of UPOs and related H_2_O_2_-dependent peroxygenases, either individually or in combination of multiple approaches. Continued integration of rational design with directed evolution and emerging machine-learning-guided strategies is expected to accelerate the development of UPOs as robust and selective catalysts for pharmaceutical synthesis.

### 3.3 AI-guided peroxygenase engineering: emerging opportunities and future directions

Directed evolution and rational design have delivered meaningful improvements in peroxygenase catalysis, yet both approaches face inherent limitations when navigating the vast combinatorial space of protein sequences. AI is emerging as a complementary tool in protein engineering and offers advantages over conventional methods because it can facilitate more efficient exploration of sequence space, thereby reducing experimental time, effort, and costs.^115,116^ Machine learning (ML), a subset of AI, uses data-driven models that make predictions for unseen data by employing numerical encoding of input and output variables, which defines their relationships without explicitly specifying them.^117,118^ The broader application of peroxygenases continues to be limited by several challenges (discussed in Section 3). The multidimensional nature of these engineering objectives, including the need to improve catalytic activity, thermostability, H_2_O_2_ tolerance, heterologous expression, substrate scope, regioselectivity, and enantioselectivity, make peroxygenases promising candidates for AI-driven engineering strategies. However, direct experimental demonstrations of AI-driven optimization of peroxygenases remain scarce to date. AI should therefore currently be considered more of an emerging complementary approach than an established development paradigm for this enzyme family.

A typical ML-assisted protein engineering workflow involves data acquisition and preprocessing, selection of suitable protein representations, model training and validation, and prediction of promising variants. Inputs to ML models can include sequence-based physicochemical descriptors, structural and evolutionary features, embeddings from pretrained protein language models (PLMs), and, where relevant, substrate-related descriptors, either individually or in combination. The potential value of integrating multiple protein representations has recently been demonstrated for CYP450 identification, where a multimodal deep-learning framework combining sequence information, PLM embeddings, and evolutionary features achieved high classification accuracy across diverse CYP450 datasets.^119^ By integrating sequence, structural, biochemical, and experimental data, ML models can learn relationships between mutations and functional properties, and prioritize variants for experimental characterization.^120^ The growth of ML-assisted protein engineering is supported by advances in gene sequencing, high-throughput screening, and the increasing availability of biological databases (BRENDA, UniProt, and PDB).^121–125^ Leveraging such data, models such as AlphaFold2 have revolutionized protein structure prediction, providing structural insights that support protein design and expand opportunities in biocatalysis and protein engineering.^126,127^

A major challenge for AI-driven peroxygenase engineering is the limited availability of systematically compiled sequence-function data. ML strategies can generally rely on supervised learning, in which models are trained using labelled datasets (such as sequence–function or structure–function relationships),^128,129^ or on unsupervised/self-supervised learning which uses large, unlabeled datasets to identify hidden patterns in protein sequences or structures.^130,131^ For peroxygenases, extensive application of fully supervised approaches is currently constrained due to limited availability of large and consistently measured sequence–function datasets. Although thousands of putative UPO sequences are available and recent studies have begun to generate larger mutational datasets, experimentally characterized sequence space remains comparatively limited, and standardized datasets suitable for supervised learning are not yet widely available across UPOs and P450 peroxygenases. This makes data-efficient inference strategies particularly attractive. Zero-shot approaches apply the pretrained models, such as PLMs, to new protein/enzyme without requiring task-specific labelled data, providing quick guidance when experimental data are scarce.^132,133^ The experimental potential of this strategy was recently demonstrated for a Baeyer–Villiger monooxygenase, where zero-shot PLM (SaProt) narrowed the experimental search to 60 candidates and ultimately led to a variant with 4.1-fold higher catalytic activity.^134,135^ Where small, characterized and labelled datasets are available, few-shot, supervised fine-tuning or transfer learning approaches can adapt pre-trained models towards specific protein engineering objectives.^136^ A study by Zhai *et al.* demonstrated that fine-tuning a general enzyme–substrate *k*_cat_ prediction framework using a P450-specific dataset led to improved prediction performance for this enzyme family.^137^ Another complementary strategy is active learning or Bayesian optimization, in which computational prediction and experimental validation are coupled iteratively. The model proposes a limited set of variants for experimental characterization, and the resulting data are used to update the model and guide subsequent design cycles, enabling efficient exploration of fitness landscapes with fewer experiments than traditional protein engineering approaches.^129,138,139^ Such iterative design–build–test–learn workflows may be particularly valuable for multi-objective optimization of peroxygenases. By prioritizing variants predicted to occupy informative or high-performing regions of the fitness landscape, these approaches can focus experimentally demanding assays on the most promising candidates rather than screening large libraries indiscriminately.

Several studies on related enzyme systems have already demonstrated methodological approaches that may be applicable to peroxygenases. For instance, Gaussian process regression and classification using structure-based kernels improved P450 chimera thermostability by 8.7 °C,^129^ while ML combining principal component analysis and *k*-means clustering from MD data guided improved *O*-demethylation selectivity of P450s for lignin bioconversion.^140^ CYP-specific deep learning frameworks provide another relevant direction. CYPMol integrates protein features derived from PLM, functionally relevant CYP residues, and pretrained small-molecule embeddings to predict CYP substrates, inhibitors and bonds of metabolism for various CYPs.^141^ It illustrates the potential of enzyme–substrate models for predicting substrate compatibility and sites of oxidation. More recently, improved variants of P450 GbCYP90J6 were identified using EVOLVEpro-based PLM-guided directed evolution employing iterative prediction–testing–retraining workflow, with the best triple mutant exhibiting an 11.39-fold increase in catalytic activity.^142^

Deep-learning-guided virtual mutagenesis combining ancestral sequence reconstruction (ASR)-based hotspot identification with DLKcat-guided screening identified valencene-hydroxylating P450 variants with up to 2.3-fold higher catalytic activity and a strong correlation between predicted and measured *k*_cat_ values.^143^ MODIFY, an ML algorithm, was experimentally applied to *Rhodothermus marinus* cytochrome *c* (*Rma* cyt *c*), where the ML-designed library identified variants capable of enantioselective C–B and C–Si bond formation *via* a new-to-nature carbene transfer mechanism.^144^ Given the shared heme-thiolate catalytic chemistry and overlapping substrate and reaction scopes of peroxygenases and P450s, P450 datasets may provide a valuable source of prior information for transfer learning in peroxygenase engineering, although transferability from conventional P450s to structurally distinct UPOs remains to be established experimentally. ML has also demonstrated its potential for improving enzyme stereoselectivity, a property quite relevant for pharmaceutical biocatalysis.^145–147^ More broadly, ML-assisted approaches have been explored for improving catalytic activity, stability, substrate specificity, and other enzyme properties, with representative examples summarized in Table 6. These studies should therefore be viewed primarily as transferable methodological precedents rather than direct evidence for established AI-guided peroxygenase engineering.

**Table 6:** Representative AI/ML-assisted protein-engineering strategies relevant to peroxygenase engineering. Studies were selected based on their direct application to peroxygenases, mechanistic relevance to heme- and oxygenase-catalyzed reactions, or demonstration of AI/ML approaches addressing engineering objectives relevant to peroxygenases. The table is intended to be representative rather than exhaustive. Preprint studies are explicitly indicated

Protein/enzyme	AI/ML method	Main outcome	Relevance to peroxygenase engineering	References
CYP152 P450 peroxygenases (OleT family)	Unsupervised ML analysis of MD trajectories using PCA and HDBSCAN, integrated with structural analysis and mutagenesis	Coordinated conformational changes in the active site were identified that control substrate positioning and chemoselectivity	Direct peroxygenase application	148
Bacterial UPO-like enzymes	Deep-learning-assisted enzyme mining	Identification and characterization of bacterial UPO-like enzymes suitable for heterologous expression in *E. coli*	Direct UPO application (preprint)	70
*Aspergillus brasiliensis* UPO (*Abr*UPO)	Task-specific LSTM within a variational search distributions framework coupled with ultrahigh-throughput screening	Generated variants with improved styrene oxide production and substrate specificity while reducing side-product formation	Direct UPO engineering (preprint)	149
P450 GbCYP90J6	PLM-guided iterative directed evolution (EVOLVEpro)	Best model-predicted variant showed an 11.39-fold improvement in catalytic activity relative to WT	Related P450 precedent; experimentally validated	142
Valencene-hydroxylating P450	Active-site analysis combined with DLKcat-guided virtual mutagenesis	Predicted variant showed 2.3-fold higher catalytic activity; predicted and measured *k*_cat_ values showed strong correlation (Pearson coefficient of ∼0.86)	Related P450 precedent; experimentally validated	143
CYP450 fusion protein CYPLY	DLKcat combined with conservation analysis and MD-guided selection of substrate-tunnel/electron-transfer residues	Predicted variants demonstrated alleviated substrate inhibition (up to 34-fold) and higher conversion rates	Related P450 precedent; experimentally validated	161
P450 CYP116B3	PyPEF using PLS and MLP regression for substrate-channel variants, followed by MD simulations	Top-ranked variant was predicted to have ∼15-fold higher activity	Highly relevant to substrate-tunnel engineering	163
P450 chimeras	Gaussian-process regression/classification using structure-based kernels	Thermostability improvement of up to 8.7 °C in *T*_50_	Related P450 precedent; experimentally validated	129
*Rhodothermus marinus* cytochrome c	MODIFY framework: ensemble PLM and sequence-density models together with Pareto optimization	Engineered variants supporting enantioselective C–B and C–Si bond formation *via* new-to-nature carbene transfer mechanism	Related heme-protein precedent; experimentally validated	144
*Rhodothermus marinus* nitric oxide dioxygenase (*Rma* NOD)	Machine learning-guided directed evolution (panel of models including linear, kernel, ensemble, and shallow neural networks)	Engineered stereo-divergent enzymes for carbene Si–H insertion (93% ee (*S*) and 79% ee (*R*))	Related heme-protein precedent	147
*Pyrobaculum arsenaticum* protoglobin (*Par*Pgb)	Active learning and Bayesian optimization using DNN ensembles with uncertainty-based acquisition	Cyclopropanation yield increased from approximately 12% to 93%	Related heme-protein precedent; experimentally validated	138
Redesigned myoglobin/heme protein (bitMb)	RFdiffusion-based backbone generation followed by ProteinMPNN sequence design and structure prediction using AlphaFold/OmegaFold	Retained heme binding/catalytic competence; *T*_m_ increased by ∼5.5 °C together with high organic-solvent tolerance	Related heme-protein generative-design precedent; experimentally validated	160
Fe(ii)/α-ketoglutarate-dependent oxygenases	ProteinMPNN-based stabilization followed by directed evolution	Improved scaffold stability, expression and evolvability toward new catalytic function	Related oxidative-enzyme precedent; experimentally validated	164
Baeyer–Villiger monooxygenase	Zero-shot SaProt-650M mutation scoring using sequence and structural information	Experimental testing of 60 prioritized mutations led to a combinatorial variant with 4.1-fold higher activity; demonstrated at 50 L scale	Transferable oxygenase precedent; experimentally validated and scale-relevant	135
4-Vinylguaiacol oxygenase	Fine-tuned ODM and trans models for distal-site prediction, integrated with catalytic-pocket engineering and MD simulations	Best variant showed ∼6.8-fold higher *k*_cat_/*K*_m_ and enabled 19.3 g L^−1^ vanillin production	Transferable oxygenase precedent; experimentally validated	165
Lysine dioxygenase (LDO)	MutComputeX integrated with MD-assisted rational design	TTN improved by ∼40%, together with increased expression	Related oxidative-enzyme precedent; relevant to TTN and expression	166
WelO5* non-heme iron enzyme	Gaussian-process regression and random forest models using amino-acid descriptor	Expanded substrate scope; ∼90-fold *k*_cat_ and ∼300-fold TTN improvement	Strong oxidative-enzyme precedent for catalytic activity, TTN and substrate-scope engineering	167
Carotenoid cleavage oxygenases (CSO2/SsCSO)	CataPro using ProtT5-XL-UniRef50 embeddings and molecular fingerprints to predict *k*_cat_, *K*_m_, and *k*_cat_/*K*_m_	Identified SsCSO with 19.5-fold higher activity than CSO2 and enabled further 3.34-fold improvement over WT SsCSO	Integration of enzyme sequence and substrate chemistry	168
*Vitreoscilla* hemoglobin (VHb)	NLP-based EESP model using bag-of-words representation and a Naive Bayes classifier	Improved enantioselectivity up to 99% ee; increased catalytic efficiency	Related heme-protein precedent for stereoselectivity engineering	169
Amidase	Random-forest classifiers trained using substrate-chemistry and 3D geometry descriptors	53-Fold increase in *E*-value for enantioselectivity	Demonstration of substrate-aware ML for stereoselectivity	170
Norbelladine 4′-*O*-methyltransferase (Nb4OMT)	MutComputeX structure-based self-supervised deep-learning model	Approximately 2-fold higher catalytic activity, an increase in *T*_m_ up to 5.6 °C, increase in product titer, and reduced side-product formation	Useful multi-property engineering precedent	171
Ketol-acid reductoisomerase (KARI)	Machine learning integrated with QM/MM simulations and multistate computational design	Variants identified with calculated *k*_cat_ increases of up to ∼20 000-fold	Integration of ML with mechanistic and QM/MM modelling	162
Artificial proteins across diverse families (including lysozyme, chorismate mutase, malate dehydrogenase, CRISPR–Cas proteins, and transposases)	ProGen and ProGen2 transformer-based autoregressive protein language models	Generated highly divergent functional protein sequences beyond naturally sampled sequence space, including experimentally validated functional enzymes and proteins	Generative-AI precedent for functional protein generation	150–153
Amide synthetase McbA	Augmented ridge regression combined with zero-shot fitness prediction	Improved enzyme activity by up to 42-fold	Zero-shot and data-efficient engineering	172
Alkane-producing enzymes/ADO-like ferritin-family proteins	ML classification using structural features, integrated with computational modelling and enzyme design	Classified structural determinants of ADO-like activity and enabled introduction of ADO-like catalytic function into a related scaffold	Relevant to oxidative enzyme discovery and design	173
Myoglobin variants	LigandMPNN ligand-aware sequence design integrated with molecular docking	Designed variants with improved conversion and altered substrate scope for cyclopropanation	Ligand-aware active-site design	146

More direct applications are beginning to emerge, although the available evidence remains limited. For CYP152 P450 peroxygenases, unsupervised ML analysis of MD trajectories was used in combination with crystallography and structure-guided mutagenesis to identify coordinated active-site conformational changes that determine substrate positioning and chemoselectivity.^148^ A recent preprint described a deep-learning-assisted enzyme mining strategy to identify bacterial UPO-like enzymes that could be heterologously expressed in *Escherichia coli*, expanding the accessible scaffold diversity beyond fungal UPOs (discussed in Section 3.1.2).^70^ Such approaches may facilitate the discovery of peroxygenases with improved expression, substrate-access architectures, or catalytic properties. Another recent preprint has also demonstrated generative sequence engineering directly for *Aspergillus brasiliensis* UPO (*Abr*UPO) by coupling ultrahigh-throughput microfluidic droplet screening with a task-specific long short-term memory (LSTM) model within the variational search distributions (VSD) framework.^149^ Trained on more than 30 000 experimentally characterized variants, the model generated *Abr*UPO sequences with improved styrene oxide production and substrate specificity, while reducing side-product formation. Although these studies represent early demonstrations rather than an established engineering paradigm, they illustrate how AI can complement high-throughput experimental screening by learning from sequence–function data to prioritize or generate promising variants. Such integrated workflows may therefore enhance, rather than replace, conventional methods.

Generative AI is opening avenues for protein engineering beyond variant prediction towards the generation of highly diverged variants and, increasingly, *de novo* protein/enzyme scaffolds. Autoregressive protein language models such as ProGen/ProGen2 have demonstrated that functional protein sequences can be generated beyond those directly sampled in nature,^150–153^ while more recent multimodal models such as ESM3 integrate sequence, structural, and functional information within a unified generative framework enabling conditional generation across multiple biological representations.^154^ Structure-conditioned sequence design tools such as ProteinMPNN^155,156^ and particularly LigandMPNN,^146,157^ which explicitly accounts for non-protein atoms such as small molecules, are highly promising for peroxygenase engineering by enabling sequence design conditioned on predefined heme and substrate geometries. All-atom generative approaches such as RFdiffusionAA and RFdiffusion2 further expand these possibilities by enabling protein scaffold generation around cofactors, ligands, and predefined catalytic geometries, thereby supporting the design of active-site environments and substrate-binding regions relevant to peroxygenases.^158,159^ The feasibility of this concept has now been demonstrated experimentally in a heme protein. Zha *et al.* reported a functional-site-scaffolding workflow combining RFdiffusion, ProteinMPNN, OmegaFold, and AlphaFold to produce a miniaturized myoglobin that retained key heme-binding features and catalytic competence while exhibiting enhanced thermal and solvent stability.^160^ For peroxygenases, such generative approaches could facilitate redesign of the heme environment, stabilization of flexible loops, improved folding, or optimization of substrate access tunnels for bulky or sterically demanding pharmaceutical substrates. At present, however, these possibilities remain predominantly prospective and require rigorous experimental validation before they can be considered established peroxygenase-engineering strategies.

Going forward, AI is unlikely to replace conventional protein engineering approaches; instead, its greatest value may lie in serving as an integrated predictive framework that combines structural models, sequence and evolutionary information, molecular docking, MD simulations, and experimental screening data with ML methods to prioritize promising mutations and guide the design of focused libraries. Recent P450 engineering provides an instructive example of why such integration is important. Zhang *et al.* showed that combining sequence-based ML with mechanistic residue selection with structural and MD analyses, led to improvement in substrate tolerance and catalytic performance.^161^ Integrating ML with mechanistic approaches, such as quantum mechanics/molecular mechanics (QM/MM) calculations, could further improve predictions of transition-state geometries, catalytic activity, and regioselectivity within peroxygenase active sites. The potential of such hybrid strategies has already been demonstrated computationally in ketol-acid reductoisomerase engineering, with calculated *k*_cat_ increases of up to approximately 20 000-fold relative to the wild type.^162^ As high-quality experimental peroxygenase datasets continue to expand, iterative active-learning strategies could identify the most informative variants for experimental evaluation, thereby generating increasingly rich sequence–function datasets and establishing a continuous feedback loop between computational prediction and experimental validation. Combined with generative and structure-aware models, these advances could enable more data-efficient, multi-objective peroxygenase engineering workflows that simultaneously optimize catalytic activity, selectivity, stability, and substrate scope, accelerating their development as robust biocatalysts for pharmaceutical synthesis.

## 4. Outlook

Peroxygenases have evolved from niche oxidative enzymes into highly adaptable catalysts capable of performing pharmaceutically relevant oxyfunctionalization reactions that are difficult to achieve using conventional synthetic chemistry. The studies reviewed here collectively suggest that the field has now reached a point at which further progress should be assessed less by isolated improvements in individual enzyme properties and more by whether engineered catalysts can satisfy the combined requirements of a defined pharmaceutical application. Accordingly, the next phase of peroxygenase research should shift from demonstrating catalytic potential toward establishing application-specific performance under preparative and ultimately process-relevant conditions.

The central lesson is that successful peroxygenase engineering increasingly depends on multidimensional optimization rather than improvement of a single catalytic parameter. The most useful biocatalyst will not necessarily be the variant with the highest activity, TTN, stability, expression level, or selectivity considered individually, but one that achieves an application-specific balance among these properties. Importantly, this balance will depend on the intended application. For synthesis of drug metabolites or analytical standards, exceptionally high selectivity at relatively small scale may justify moderate catalyst productivity, whereas manufacturing of pharmaceutical intermediates places greater emphasis on overall catalyst and process productivity. Future engineering campaigns should therefore define the target performance profile of the biocatalyst before optimization rather than treating all desirable enzyme properties as equally important.

A corresponding priority is to establish application-specific benchmarks that allow engineered peroxygenases to be evaluated against realistic alternatives. Rather than expanding the number of reported catalytic descriptors, future studies should connect molecular improvements directly to outcomes relevant to the intended use *e.g.* the amount of isolated product obtained per unit catalyst, the attainable product concentration, or the overall efficiency of a preparative transformation. Such benchmarking would make it possible to determine when an improvement in an enzyme property produces a meaningful advantage at the level of synthesis and when it merely represents a better laboratory-scale catalyst.

This application-centered perspective also implies that protein and process optimization should increasingly proceed concurrently. The central question is not whether challenges such as enzyme production, peroxide management, substrate accessibility, and operational stability can each be improved independently, the preceding studies demonstrate that they can, but which combinations of improvements are required for a particular transformation and which trade-offs become limiting as scale and substrate loading increase. Systematic mapping of these interdependencies could provide a more useful basis for engineering than continued optimization against single-property screening objectives.

Meeting these multi-objective requirements will also require greater integration of complementary engineering approaches. Table 7 lists the comparison of different protein engineering strategies in terms of their relative success probability, cost and experimental burden, transferability, and industrial applicability. Directed evolution, rational and structure-guided design, computationally aided approaches, enzyme mining, and AI/ML address different parts of the same optimization problem and should increasingly be deployed as complementary approaches rather than regarded as competing. A promising workflow would begin with selection of a naturally suitable scaffold, use structural or data-driven approaches to identify high-value sequence regions, experimentally explore focused libraries, and return the resulting quantitative data to subsequent rounds of prediction and design. In this framework, natural diversity becomes particularly valuable not simply as a source of additional peroxygenases, but as a means of identifying parental scaffolds that already possess favorable application-relevant characteristics, thereby reducing the extent of subsequent engineering required.

**Table 7:** Comparison of different protein engineering strategies. The comparison highlights that no single engineering strategy is universally optimal for peroxygenases. Directed evolution remains the most broadly transferable approach and can improve multiple properties without detailed mechanistic knowledge, but its effectiveness and cost depend strongly on the availability of suitable high-throughput screening methods. In contrast, rational approaches such as oxygen-binding-groove, substrate-pocket and H_2_O_2_-tunnel engineering can substantially reduce library size and experimental burden, although their success relies more heavily on structural and mechanistic understanding and may be less transferable between enzyme scaffolds. Computationally guided approaches can further narrow experimental search space, whereas AI/ML-guided engineering remains an emerging strategy for peroxygenases whose future impact will depend on the availability of sufficiently large and standardized sequence–function datasets. From an industrial perspective, these strategies are best regarded as complementary rather than mutually exclusive, with their combined use tailored to the specific catalytic and process bottlenecks of each application

Engineering strategy	Typical design principle/engineering scope	Representative performance reported in this review	Success probability	Cost/experimental burden	Transferability	Industrial relevance	Major advantages	Major limitations
Directed evolution	Iterative random/semi-random mutagenesis followed by high-throughput screening; explores sequence space with limited requirement for prior mechanistic knowledge	P450 peroxygenase variants: >99% ee and TTNs up to 3278; AeUPO variants SoLo: TTN up to 62 000; *Aae*UPO variant PaDa-I enabled strongly enhanced heterologous UPO expression	High, when a robust HTS assay is available	High	High	High	Broadly applicable; can optimize activity, selectivity, expression and stability simultaneously; does not require detailed structural understanding	Requires library construction and extensive screening; strongly dependent on suitable HTS assays; multiple rounds may be required
Oxygen-binding groove engineering	Rational modification of the conserved P450 I-helix/acid–alcohol pair to promote H_2_O_2_ activation and compound I formation	CYP119: >10-fold activity at RT and up to 35-fold at 80 °C; CYP154C8: >100-fold peroxygenase activity; CYP107PQ1: ∼94% ee	Moderate to high for P450s with conserved catalytic architecture	Low to moderate	Moderate to high within P450s	Moderate to high	Small number of targeted mutations; low screening burden; mechanistically interpretable; directly converts P450 monooxygenases toward H_2_O_2_-driven catalysis	Requires structural/mechanistic knowledge; primarily applicable to enzymes sharing an appropriate oxygen-binding architecture
DFSM-assisted catalysis	An exogenous dual-functional small molecule anchors in the substrate channel while providing an acid–base group for H_2_O_2_ activation	P450BM3/DFSM: TTN up to 80 963, >99% regioselectivity and >99% ee; preparative indigo synthesis reached 65.8% isolated yield	High for established P450BM3/DFSM systems	Moderate	Moderate	Moderate to high	Modular; avoids extensive protein modification; DFSM structure can tune H_2_O_2_ activation, substrate positioning and selectivity	Requires an additional small-molecule component; performance depends on compatibility among enzyme, DFSM and substrate; adds process complexity
H_2_O_2_ tunnel engineering (HTE)	Structure-/MD-guided remodeling of water/H_2_O_2_ access tunnels to improve peroxide delivery to buried heme	CYP199A4: >183-fold improvement and TTNs >1000; CYP153A: 15-fold improvement; CYP105D18: ∼80-fold higher catalytic efficiency	Moderate to high when H_2_O_2_ transport is rate-limiting	Moderate	Moderate	Moderate to high	Directly addresses mass transport of H_2_O_2_; relatively focused mutation space; can strongly enhance peroxide-shunt activity	Requires structural information and identification of relevant tunnels/gating residues; benefit may be limited when peroxide accessibility is not the major bottleneck
Active-site/substrate-binding pocket engineering	Rational or semi-rational remodeling of substrate-binding residues using structural analysis, docking and/or MD to optimize substrate orientation	*Cvi*UPO: 13-fold activity and >99% ee; ancestral UPO variant: 36-fold activity and 99% 11β-regioselectivity, enabling gram-scale steroid synthesis; *Mth*UPO: 17-fold activity and 99.6% regioselectivity	Moderate to high	Moderate	Moderate	Moderate to high	Particularly effective for tuning regio-/stereoselectivity and substrate scope; focused libraries substantially reduce screening burden	Requires structural/mechanistic knowledge; mutations can be substrate-specific; predictions of productive substrate orientation are not always reliable
Computational/structure-guided focused-library design (*e.g.*, PROSS, FuncLib, MD-guided design)	Computational identification and prioritization of mutations followed by construction of small, information-rich libraries; includes approaches such as PROSS, FuncLib and MD-guided design	PROSS: 4-fold enhanced H_2_O_2_ tolerance; FuncLib *Aae*UPO: only 24 variants, with up to 15-fold higher TTN; FuncLib *Mth*UPO: only 50 variants, with 2.2–7.1-fold activity improvement and substantial selectivity changes	Moderate to high	Low to moderate experimental cost; moderate computational cost	High, provided reliable sequence/structural information is available	Moderate to high	Greatly reduces library size and experimental screening; enables multiparameter engineering; compatible with automation and iterative design	Dependent on structural/model quality and computational predictions; may miss beneficial mutations outside the predefined design space
AI/ML-guided engineering	Data-driven prediction and prioritization of sequence–function relationships and promising variants	Direct experimental demonstrations for peroxygenases remain scarce; currently mainly an emerging complementary strategy	Emerging; limited direct experimental evidence in peroxygenases	Potentially low experimental burden; high initial data/computational requirements	Potentially high	Promising, but currently emerging	Potential to explore large sequence spaces, reduce experimental screening and support multi-objective optimization	Limited standardized peroxygenase datasets; prediction quality is data-dependent; experimental validation remains essential

For AI/ML, the most important near-term opportunity may therefore lie not in replacing established engineering strategies, but in improving the efficiency with which multi-property trade-offs are explored. Progress will depend on datasets that connect sequence and structure not only to catalytic phenotypes but also to measurements obtained under sufficiently comparable experimental conditions. Particularly valuable would be the datasets containing unsuccessful or intermediate variants alongside high-performing enzymes, because these data can define the boundaries of viable sequence–function space and reveal trade-offs that are obscured when only successful variants are reported. Active-learning approaches could then prioritize experiments according to both predicted performance and information gain, making each engineering cycle progressively more informative. In the longer term, the convergence of generative protein models, automated construction and screening, and design–build–test–learn cycles could enable increasingly closed-loop peroxygenase engineering. The important transition would not simply be toward greater automation, but toward systems in which experimental outcomes continuously redefine the engineering objective and the regions of sequence space explored. Given the limited standardized sequence-function data currently available for peroxygenases, however, this remains a longer-term direction rather than an established capability.

Another emerging direction is to consider peroxygenases as components of integrated synthetic systems rather than exclusively as isolated oxidation catalysts. Multi-enzyme cascades, controlled peroxide-generation modules, and chemoenzymatic sequences may overcome limitations that are difficult to resolve through mutation alone while enabling synthetic routes inaccessible to individual catalysts. Their significance may be greatest where integration changes the synthetic route itself *e.g.* by avoiding intermediate isolation, enabling otherwise incompatible reaction sequences, or coupling oxidation directly to upstream or downstream transformations. Consequently, future comparisons should consider whether such systems simplify the complete synthesis rather than whether they improve the peroxygenase step in isolation.

More broadly, an important opportunity for the field is to move from asking how broadly peroxygenases can oxidize pharmaceutical molecules toward identifying transformations for which they offer a distinctive synthetic advantage. This will require closer matching of enzyme capabilities to unmet synthetic needs, particularly transformations in which conventional methods struggle to achieve the required site-, chemo-, or stereoselectivity on complex molecular scaffolds. Such application-led discovery could help prioritize substrates and reactions with the greatest potential impact rather than continuing to expand substrate scope primarily as a demonstration of catalytic versatility.

Overall, the studies discussed in this review demonstrate that peroxygenases are transitioning from proof-of-concept biocatalysts toward practical oxidative tools for pharmaceutical synthesis. The critical question for the next stage of the field is no longer only whether a peroxygenase can catalyze a pharmaceutically relevant transformation, but whether the complete catalyst-reaction system can perform that transformation selectively, reproducibly, and economically at the required scale. The defining advance will therefore be a shift from enzyme-centered optimization to application-centered development, in which the desired pharmaceutical transformation determines the catalyst profile, engineering strategy, experimental metrics, and process configuration from the outset. If this transition is achieved, peroxygenases could progress from versatile laboratory oxidation catalysts to differentiated synthetic technologies for pharmaceutical development and manufacturing.

## Author contributions

L. L. F., A. K. and M. S. P. P.: conceptualization, writing – original draft, writing – review & editing, visualization; U. S.: conceptualization, writing – review & editing, funding acquisition.

## Conflicts of interest

The authors declare that they have no competing interests.

## Data Availability

No primary research results, software or code have been included and no new data were generated or analysed as part of this review.
